# Beyond Meat Substitution: A Multifaceted Review of Plant-Based and Alternative Proteins, from Environmental Impact to Analytical Technologies

**DOI:** 10.3390/foods14132312

**Published:** 2025-06-30

**Authors:** Abel Navarré, Leonardo Musto, Tiago Nazareth

**Affiliations:** 1 Department of Pharmacy, University of Naples Federico II, Via Domenico Montesano 49, 80131 Naples, Italy; abel.navarredopazo@unina.it; 2 Department of Preventive Medicine and Public Health, Food Science, Toxicology and Forensic Medicine, University of Valencia, Av. Vicent Andrés Estellés s/n, 46100 Burjassot, Spain; leonardo.musto@uv.es

**Keywords:** meat analogues, plant-based diets, sustainability, innovative ingredients, environmental impact, sensory acceptance, electronic sensors, edible insects, cultured meat, microalgae

## Abstract

The escalating environmental and health concerns regarding conventional meat consumption have intensified the global search for sustainable dietary alternatives. Plant-based foods and meat substitutes have emerged as promising solutions. These products aim to replicate the sensory and nutritional attributes of meat while mitigating ecological impacts. This review examined the current scenario of plant-based foods and meat substitutes, focusing on their environmental footprints, health implications, innovative ingredient developments, consumer acceptance, and the use of analytical tools in quality control. Life cycle assessments indicate that plant-based foods and meat substitutes significantly reduce greenhouse gas emissions, land use, and water consumption compared to animal-based products. These alternatives offer benefits like lower saturated fat. However, they still struggle to match the amino acid composition of meat. Consumer acceptance is influenced by factors including taste, texture, and cultural perceptions, and still requires sensory improvement. Innovations in ingredient sourcing, like the use of legumes, mycoproteins, and fermentation-derived components, are enhancing product quality and diversity. Furthermore, analytical tools such as electronic noses, electronic tongues, spectroscopy, and chemometric methods ensure product consistency and fulfill consumer expectations. By synthesizing interdisciplinary insights, this review offers an integrated perspective to guide future research and development in the field of meat alternatives.

## 1. Introduction

The global food system is under growing pressure due to its environmental, ethical, and public health impacts. Livestock production is one of the most resource-intensive sectors. It contributes significantly to greenhouse gas (GHG) emissions, deforestation, water use, and biodiversity loss. Life cycle assessments (LCAs) have consistently demonstrated that animal-based foods, especially red meat from ruminants, carry a much higher environmental footprint than their plant-based counterparts. In 2018, global agriculture produced approximately 12% of total GHG emissions (excluding land-use change and forestry), making it the second-largest emitter after the energy sector. Overall, the food system as a whole accounted for about 21–37% of emissions, with meat consumption being a significant contributor [1]. Livestock alone contributes around 14.5% of anthropogenic GHGs and uses nearly 30% of the world’s freshwater [2]. With the global population projected to reach 9 billion by 2050 and demand for animal-source foods expected to double, sustainable dietary alternatives have become urgent [3].

To limit global warming to 1.5 °C, reducing agriculture’s land footprint is essential. This can be achieved by improving production efficiency and encouraging dietary changes, particularly shifting away from ruminant meat and toward plant-based foods [4]. Simultaneously, high consumption of animal-derived products has been associated with adverse health outcomes, including cardiovascular diseases, certain types of cancer, and increased all-cause mortality [5,6].

In response to these challenges, the development of plant-based foods (PBFs) and meat substitutes (MSs) has gained attention as promising strategies to promote sustainable and health-conscious dietary patterns. While PBFs encompass a broad category of foods derived from plant ingredients, MSs refer to specific products that are designed to replicate the sensory, nutritional, and functional properties of conventional meat. These products aim to reduce ecological impact and meet consumer expectations; however, their effectiveness is moderated by issues such as ultra-processing, nutrient deficiencies, and environmental costs of production [7]. Moreover, their effectiveness depends not only on environmental and health metrics but also on the integrity of their formulation. To truly support healthy and sustainable diets, these alternatives must avoid excessive ultra-processing and provide adequate nutrition.

The market for plant-based meat alternatives has experienced significant growth in recent years, driven by increasing consumer interest in health, environmental, and ethical considerations. Additionally, governmental policies and regulatory frameworks are shaping the market by implementing incentives for sustainable food production, developing labeling regulations, and promoting dietary guidelines that encourage plant-based diets [8]. However, widespread acceptance remains limited, particularly among omnivores and flexitarians, who are often discouraged by unfamiliar textures, flavors, and concerns about high levels of processing. Although products such as tofu and tempeh have long been accepted by vegan and vegetarian consumers, their adoption among meat-eaters has been limited due to poor sensory appeal. Moreover, plant proteins used in MSs often lead to undesirable sensory attributes, such as beany or grassy off-flavors, and tend to lack the juiciness and fibrous structure of meat, mainly due to differences in fat content and emulsifying capacity. As a result, achieving consumer satisfaction requires technological innovation and targeted sensorial optimization [9].

In light of these limitations, some researchers have questioned whether technological and analytical advances alone can replace the structural, emotional, and cultural roles that meat plays in the human diet. The discrepancy between consumer intentions and actual purchasing behavior—especially when expectations regarding taste and nutrition are unmet—suggests a more complex interplay between familiarity, perception, and performance.

Although awareness of MSs is increasing, many products still fail to achieve widespread acceptance. They often lack the sensory familiarity and cultural embeddedness of traditional meat [10]. While technology may improve mimicry, it does not guarantee social acceptance [11]. Full substitution of meat remains a debated and perhaps unrealistic short-term goal. A more pragmatic strategy might involve hybrid formulations that combine animal and plant proteins to better meet sensory and sustainability expectations.

So far, previous reviews have studied plant-based diets and MSs, but each one focuses on a single topic [12,13,14,15]. For example, Kustar and Patino-Echeverri [15] reviewed the environmental impact of these products, showing that they can reduce emissions and land use. However, they did not discuss nutrition or product quality. López-Moreno and Kraselnik [12] focused on how plant-based proteins affect muscle strength and body composition. Their review explained the nutritional differences between plant and animal proteins, but they did not consider sustainability or processing. Nowakowski et al. [14] studied how consumers react to new protein sources. They discussed acceptance and willingness to try alternatives, but did not include analytical methods or food technology. So far, no review has combined all these perspectives in one paper [12,13,14,15]. Integrated, critical evaluations that also incorporate consumer acceptance and analytical quality control remain limited. In particular, the role of emerging analytical tools, such as electronic noses (e-nose), electronic tongues (e-tongue), spectroscopy, and chemometric techniques, has received little systematic attention, despite their growing relevance in optimizing formulation, standardizing sensory profiles, and ensuring product quality [16].

To address these gaps, this review evaluates current evidence on plant-based meat substitutes (PBMs) from a multidimensional perspective. It focuses on (i) the environmental and health implications of replacing conventional meat with PBFs and MSs, (ii) innovations in ingredient sourcing and formulation, (iii) consumer acceptance of sensory aspects, and (iv) the application of advanced analytical methods to assess sensory and physicochemical properties. This review includes sustainable meat alternatives such as PBFs, MSs, cultured meat, hybrid products, and insect-derived proteins intended for human consumption. By integrating insights from sustainability science, nutrition, food technology, and sensory analytics, we aim to offer a comprehensive foundation to guide future development and evaluation of alternative protein sources.

## 2. Methodology for Literature Selection

In alignment with PRISMA guidelines [17], the literature selection process for this review followed a systematic strategy to identify, assess, and synthesize scientific articles on PBFs and MSs. Scopus and PubMed databases were queried for the period 2020–2025 using tailored search strings according to five thematic areas: (1) sustainability and environmental impact, (2) health outcomes of plant- vs. animal-based dietary patterns, (3) innovative ingredients and environmental feasibility of future meat alternatives, (4) sensory acceptance and consumer trends, and (5) analytical tools used for quality evaluation of PBFs and MSs.

Among the selected databases, Scopus provides extensive coverage of scientific literature across diverse areas, including sustainability and food science and technology, while PubMed was selected as the complementary database due to its distinct coverage, retrieving documents not captured by Scopus, particularly those related to human health. Other comprehensive databases, such as Web of Science, were excluded after a preliminary assessment confirmed that they yielded no additional relevant results beyond those obtained through the combined Scopus and PubMed search strategy. The search was limited to studies published within the last five years to capture the most current literature, given that the examined topics represent rapidly evolving fields that have undergone significant developments in recent years. Key reviews within each thematic domain served as references to optimize keyword selection and ensure comprehensive search coverage.

Search terms were tailored for each thematic block. For sustainability and environmental impact, the search combined the terms “environmental impact” or “sustainability” with “plant-based” and “meat substitute” or “meat analogue”, yielding 98 results in SCOPUS and 16 in PUBMED. Regarding the health implications of dietary patterns, the search was refined to the abstract field using the terms “plant-based” and “pattern” along with “all-cause” and “mortality”. This strategy retrieved 36 articles from PUBMED and 27 from SCOPUS. Articles were retained if they compared predominantly plant-based and animal-based diets, focused on prospective cohort studies, and used validated dietary indices such as the overall, healthy, or unhealthy plant-based diet index (oPDI, hPDI, uPDI). After screening for relevance, a total of 11 studies were included in this section of the review.

To explore the feasibility and environmental profile of novel ingredients, multiple parallel searches were performed using combinations of “life cycle assessment” with specific protein sources. For edible insects, the keywords included insect-related terms such as “cricket”, “mealworm”, and “locust”, resulting in 24 records in SCOPUS and 13 in PUBMED. After reviewing abstracts and removing duplicates, 20 articles were retained. For cultured meat, the terms “cultured meat” and “artificial meat” were used alongside “life cycle assessment”, leading to the inclusion of 12 studies after an initial pool of 25. Similarly, 7 studies on mycoprotein and several others on microalgae, such as *Spirulina* and *Chlorella*, were identified and selected based on their focus on sustainability and food applications.

The section on consumer sensory perception and market trends involved a search using the terms “sensorial”, “meat”, “consumer”, and “substitute” or “alternative”. A total of 44 articles were retrieved from SCOPUS. After abstract screening, only 4 studies were retained, of which 2 were included based on their methodology: they had to compare 100% plant-based products with animal-based counterparts and include sensory assessments from untrained consumer panels.

For the analytical assessment of MS and PBF, the query combined “plant-based” or “meat-substitute” with terms like “electronic noses”, “electronic tongues”, “sensors”, “chemometrics”, or “pattern recognition”. Around 80 articles were identified, and following abstract screening and relevance verification, a subset of 12 was included based on their application of electronic sensors for evaluating physicochemical or sensory properties.

All retrieved articles were screened manually in a two-step process: first, titles and abstracts were manually reviewed for relevance and compliance with inclusion criteria; then, full texts were examined for methodological robustness and alignment with the thematic focus. Only original research articles written in English and published between 2020 and 2025 in peer-reviewed journals were considered. Review articles, conference abstracts, editorials, and papers focusing on non-food applications or animal feed were excluded. After duplicate removal and detailed screening, 63 articles were finally included and categorized according to their thematic relevance.

Inclusion criteria required studies to (i) be original research articles, (ii) focus on PBF or MS in the context of health, environmental impact, technology, or analytical evaluation, and (iii) be published in peer-reviewed journals in English. Exclusion criteria included review articles (unless deemed highly relevant to the topic), editorials, and non-English-language papers. Duplicates were removed, and the remaining titles and abstracts were screened for relevance, followed by full-text analysis.

Data extraction focused on study design, sample population, environmental and nutritional outcomes, sensory analyses, and analytical methodologies. Furthermore, a snowballing technique was performed as relevant articles were found and clusters of thematic emphasis were identified across the included articles.

**Figure 1:**
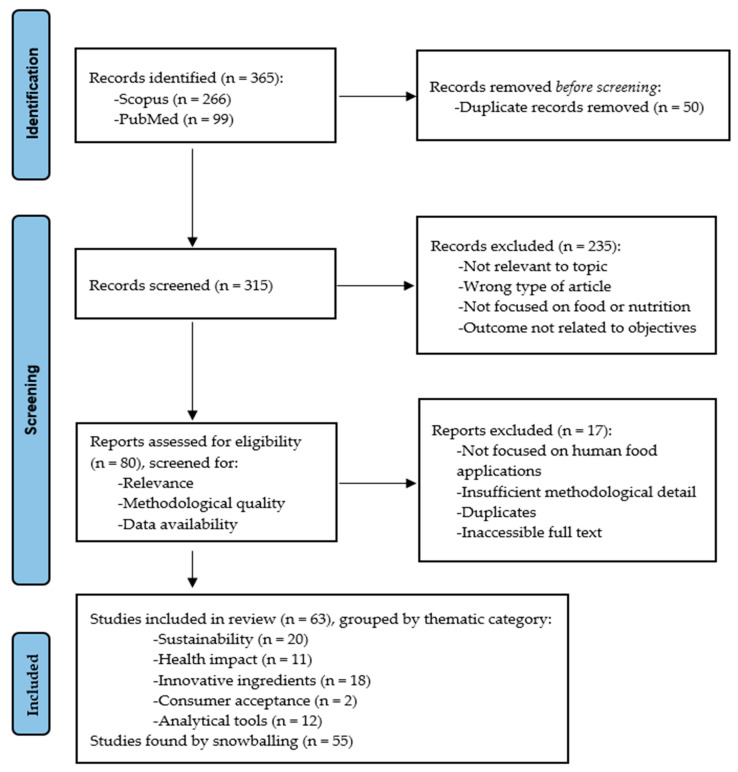
PRISMA flow chart of literature selection for systematic review.

This methodology enabled a rigorous and thematically structured synthesis of the current literature on plant-based meat alternatives, highlighting environmental, nutritional, sensory, and technological dimensions. The selection process is summarized graphically in the PRISMA flow chart presented in Figure 1.

## 3. Results of PRISMA Search

### 3.1. Sustainability and the Environmental Impact of PBF Versus Meat

The environmental ramifications of food production are increasingly recognized as central to the transition toward sustainable food systems. At the core of this discourse lies the comparative evaluation of PBF and meat, a subject of substantial academic scrutiny and public debate. Proponents of plant-based diets highlight their lower GHG emissions, reduced resource utilization, and comparatively minimal ecological disturbance. Conversely, critics underscore the challenges posed by industrial-scale processing, regional agricultural disparities, and the complex nutritional trade-offs inherent in such dietary transitions.

#### 3.1.1. Favorable Environmental Impacts of Plant-Based Diets

The cultivation of legumes, grains, and other plant-based crops plays a transformative role in reducing dependency on land-intensive livestock farming. This shift mitigates deforestation and safeguards carbon sinks such as forests, which are often converted into pastures or cropland for animal feed [18]. Such reductions in land-use change also yield significant biodiversity conservation benefits. Moreover, replacing traditional livestock farming with plant-based agriculture significantly reduces methane emissions, a potent GHG, from ruminants.

While both García-Vega et al. [19] and Harwatt et al. [18] differ in scope and approach, they both emphasize the importance of sustainable agricultural practices such as reducing land farming and present valuable points of view. García-Vega et al. [19] focus on establishing a “safe agricultural space for biodiversity”, exploring the tensions between agricultural production and biodiversity conservation. They explored the complexities of land sharing and sparing, ecosystem services, and regenerative agriculture, providing a nuanced perspective on how to minimize agriculture’s negative impacts on biodiversity. In contrast, Harwatt et al. [18] adopted a broader lens, examining the environmental impacts of food production and consumption within the Nordic and Baltic regions. While acknowledging the importance of biodiversity, the report prioritizes dietary shifts towards plant-based diets and reductions in food waste as key strategies for minimizing environmental burdens. This difference in focus highlights the multifaceted nature of sustainable food systems, requiring consideration of both production practices and consumption patterns to achieve true sustainability.

Plant-based agricultural systems require markedly fewer inputs of nitrogen and phosphorus compared to livestock farming, thereby curbing nutrient runoff into aquatic ecosystems. This mitigates eutrophication—a critical environmental issue exacerbated by intensive animal farming systems [20]. Additionally, plant-based farming systems can leverage crop rotation techniques to enhance soil fertility and reduce dependency on synthetic fertilizers, further curbing environmental degradation.

This reduced reliance on synthetic fertilizers, a hallmark of plant-based systems, offers a stark contrast to conventional agriculture’s heavy dependence on these inputs. The environmental consequences of this dependence are multifaceted. Synthetic fertilizer production is energy-intensive, contributing to GHG emissions [18]. Over-application of these fertilizers leads to nutrient runoff, polluting waterways and fueling harmful algal blooms [18]. Furthermore, long-term use of synthetic fertilizers can degrade soil health, disrupting microbial communities and reducing soil organic matter [21]. Blakstad et al. [20] underscore the importance of considering local contexts when evaluating the potential of plant-based systems. While the study acknowledges the environmental benefits of plant-based protein sources, it also highlights the challenges of achieving nutritional adequacy in resource-constrained contexts, emphasizing the need for integrated strategies that encompass both environmental sustainability and the social dimensions of food security.

LCAs consistently underline the environmental advantages of PBFs. Marquardt et al. [22] conducted a prospective LCA comparing plant-forward and meat-based dietary options in Germany and Indonesia, demonstrating that plant-forward diets exhibit significantly lower GHG emissions across both present and future production scenarios. These findings illustrate the potential of plant-based diets to reduce carbon footprints while mitigating climate change impacts. While Marquardt et al. [22] offered compelling evidence for the lower GHG emissions of plant-forward diets, several limitations warrant consideration. The authors themselves acknowledge the inherent uncertainties in prospective LCA methodologies, particularly when projecting future impacts based on Shared Socioeconomic Pathways (SSPs) [22]. The chosen SSPs, while representing plausible future scenarios, may not fully capture the complex interplay of factors influencing dietary choices and agricultural practices. How sensitive are the results to variations in these pathways? Further, the study’s comparison of plant-forward meals to a generalized “meat-based” diet lacks specificity [22]. A more detailed characterization of the meat-based diet’s composition, considering regional variations in meat consumption patterns, would strengthen the analysis. This lack of specificity limits the generalizability of the findings, making it challenging to translate the results into concrete dietary recommendations.

Comparing Marquardt et al.’s [22] findings with other LCA studies reveals both consistencies and discrepancies. Kim et al. [23], examining dietary patterns in the United States, provide further evidence of the environmental benefits of plant-based diets. Their full food supply chain perspective [23] corroborates the importance of considering the entire life cycle of food products, not just the consumption phase. However, Auclair et al. [24], exploring protein substitutions in Canadian diets, highlight the complex trade-offs between nutritional, health, and climate outcomes. Their work [24] underscores the need for tailored strategies that balance environmental sustainability with nutritional adequacy. Mazac et al. [25], focusing on novel foods, present yet another perspective, emphasizing the potential for innovative food technologies to contribute to more sustainable diets. These varied approaches to dietary LCAs [23,24,25] demonstrate the complexity of evaluating food systems and the importance of considering multiple perspectives.

Plant-based systems generally require less land and water than livestock farming, particularly in protein-dense crops like legumes and cereals, a factor that is especially relevant in regions facing resource constraints [20]. Transitioning to plant-based proteins can also alleviate pressures on biodiversity hotspots endangered by livestock-driven deforestation [19,22].

However, these benefits are context-dependent, varying with crop type, irrigation methods, and fertilizer use, which can significantly alter environmental outcomes. For instance, Blakstad et al. [20] assert that legumes and cereals require substantially less water and arable land per unit of protein compared to beef and other meats. The specific data and methodologies underlying these comparisons require further scrutiny, and some questions should be clarified, such as the following: In what contexts were these comparisons made? Do they account for variations in agricultural practices, such as irrigation methods or fertilizer use, which can significantly influence water and land requirements? This factor is of critical importance in regions grappling with water scarcity and limited agricultural resources, as highlighted by Blakstad et al. [20], who discuss the strain on land and water resources due to increased protein demands in Addis Ababa.

However, even within plant-based systems, resource use varies significantly. For example, some crops, like almonds, can have high water footprints despite being plant-based [18]. Therefore, broad generalizations about resource efficiency should be nuanced with specific examples and data [18]. Furthermore, the assertion that transitioning to plant-based proteins can alleviate pressures on biodiversity hotspots endangered by livestock-driven deforestation requires further examination since even the use of pesticides in plant-based production can alter insect fauna [22]. Livestock agriculture is undoubtedly claimed as a major driver of deforestation, yet other factors, such as agricultural expansion for crop production, also make a significant contribution [18,22]. A more nuanced analysis would acknowledge these complexities and explore the potential trade-offs and unintended consequences of different agricultural practices.

The interaction between environmental benefits and consumer behavior warrants careful consideration. Effective promotion of plant-based diets necessitates addressing dietary preferences, enhancing economic accessibility, and bolstering nutritional education to drive widespread adoption. Policy interventions that support local plant-based agriculture have the potential to advance sustainability goals while simultaneously bolstering food security and regional economic development [20,26]. For instance, initiatives incentivizing smallholder farmers to cultivate protein-rich legumes can ensure a steady supply of PBFs while creating equitable economic opportunities.

Moreover, the economic and social ramifications of dietary shifts deserve careful consideration. Potential job displacement in the animal agriculture sector [27], coupled with the cultural importance of animal-sourced foods in certain communities [28], requires nuanced analysis. Understanding how changes in food production impact livelihoods and food security is crucial for developing just and sustainable transitions. For example, linking ecosystem service provisioning with the demand for animal-sourced food [29] provided insights into the complex interdependencies between environmental sustainability and economic resilience. The study of sustainable diets in developing countries like Vietnam and Kenya [30] underscored the necessity of conducting context-specific environmental analyses to inform targeted interventions.

It is important to emphasize that assessing the environmental impact of dietary changes demands a multidisciplinary approach that considers total impact, not just individual metrics. While plant-based diets generally exhibit lower environmental footprints [31], examining the full range of environmental factors, including land and water use, GHG emissions, and biodiversity impacts, remains essential [32]. Failing to consider the totality of environmental effects could lead to overlooking potential trade-offs and unintended consequences [33]. Furthermore, recognizing the stages of dietary transitions and the context-specific nutritional and health outcomes associated with varying dietary patterns [24,34] is crucial for developing effective strategies.

A critical analysis of the transition’s economic and social impacts must consider how interventions influence different demographic groups [35]. Moreover, the importance of culturally acceptable, affordable, and easily implemented meal replacements is underscored by the potential nutritional gaps arising from transitions to lower-impact options [22]. Recognizing the heterogeneity of motivations for adopting plant-based diets, including ethical concerns and health considerations, can inform the design of educational programs and targeted incentives. For instance, novel PBFs may play a significant role [26,36], and addressing consumers’ practical constraints, including nutritional “leakage” from meal substitutions, is crucial for facilitating successful transitions and avoiding generating other negative consequences.

#### 3.1.2. Challenges and Critiques of PBFs

Although plant-based proteins offer numerous environmental benefits, their production and consumption are not exempt from challenges. Soy, a predominant source of plant-based proteins, exemplifies this complexity. Monoculture farming of soybeans, particularly in South America (like in Brazil, the world’s biggest soybean producer), has been linked to deforestation, soil degradation, and excessive pesticide use. Despite its high protein yield per hectare, the environmental footprint of soy cultivation raises concerns about scalability without exacerbating ecological problems [37]. Additionally, soy farming often displaces indigenous communities and disrupts local ecosystems, highlighting the socio-environmental costs of reliance on a single crop in a process called “agriculturalization”.

For example, soybean crops occupy a significant portion of Brazil’s territory, with production having expanded considerably since 1990 by over 313% [38]. While the Amazon rainforest often receives more attention, the Cerrado biome is the primary region for soybean cultivation in Brazil [39]. This biome, a biodiversity hotspot, faces substantial environmental pressures from agricultural expansion, including deforestation and threats to numerous plant and animal species [35]. Soybean production contributes significantly to GHG emissions in Brazil [40]. Furthermore, the extensive use of pesticides in soybean farming raises concerns about water, soil, and food contamination, and potential human health risks [41]. This complex situation underscores the need to balance the benefits of soy protein as a readily available plant-based protein source in countries with the environmental and social costs associated with its production. The intensification of existing agricultural lands, including improving soybean yields through techniques like inoculation [42], presents a more sustainable pathway compared to further expansion into natural habitats [43]. This approach aligns with efforts to protect valuable ecosystems like the Cerrado and Amazon while meeting the increasing demand for plant-based protein [44]. Additionally, exploring alternative production systems and incorporating sustainable practices, such as crop rotation and minimizing soil disturbance, can help mitigate the negative impacts of soybean cultivation [45]. LCAs that consider land-use change, transportation, and other factors provide a complex view of the environmental impact of soybean-based products, enabling informed decision-making about sustainable sourcing and consumption [46]. Therefore, a transition towards more sustainable monoculture production is crucial not only for countries that base their economy on agriculture but also for the global food system.

Reliance on imported PBFs can further complicate their environmental advantages. For regions unable to produce sufficient quantities locally, transportation emissions from imports like quinoa, avocados, and almonds significantly elevate the carbon footprint. Balancing local production with global trade is essential to preserving the net environmental benefits of these foods [47]. Moreover, the heavy reliance on water-intensive crops such as almonds raises questions about the sustainability of their large-scale production in drought-prone areas.

Socioeconomic disparities also emerge as a critical consideration in the adoption of plant-based diets. Novel plant-based products, particularly those marketed as premium alternatives, often come at higher price points than traditional staple foods. This economic barrier limits accessibility for low-income populations. Furthermore, cultural preferences and culinary traditions can hinder the widespread adoption of plant-based diets in regions where meat consumption is deeply ingrained in social identity [26]. Efforts to mainstream PBFs must therefore address both affordability and cultural adaptability to ensure equitable dietary transitions.

Processing of PBFs introduces additional complexities. The industrial production of ultra-processed plant-based products, such as protein isolates and MSs, often requires substantial energy and water inputs. These demands can approximate the environmental burdens associated with minimally processed meats, underscoring the necessity for in-depth supply chain evaluations to avoid unintended trade-offs [47]. Furthermore, the reliance on additives and stabilizers in these products raises health concerns that merit further investigation.

Moreover, many LCAs fail to account for critical stages in the life cycle of plant-based proteins. These stages encompass a range of processes, including extraction, concentration, and purification of protein isolates, all of which require substantial energy inputs and the use of highly specialized equipment. For instance, protein extraction often involves solvent-based methods or mechanical separation techniques that demand significant operational resources and generate secondary waste products. Similarly, protein concentration and purification steps necessitate advanced filtration or chromatographic systems that further contribute to the overall environmental footprint. The cumulative energy consumption and reliance on non-renewable inputs in these processes raise critical questions about the scalability and long-term sustainability of plant-based protein production. Addressing these inefficiencies through innovations in processing technologies, such as low-energy extraction methods or closed-loop systems, is essential to reducing the environmental impact while maintaining the functional and nutritional quality of these proteins [37].

Nutritional and social trade-offs present further challenges. Although plant-based diets are broadly regarded as environmentally sustainable, concerns about nutritional adequacy persist, particularly for populations with elevated protein requirements. Nájera Espinosa et al. [26] observed considerable variability in the nutrient profiles of plant-based alternatives, with some products exhibiting deficiencies in essential micronutrients like iron and vitamin B_12_. These gaps pose challenges for regions with limited dietary diversity. Furthermore, emerging evidence suggests that the bioavailability of certain nutrients in PBF may be lower than their animal-derived counterparts, necessitating fortification or supplementation to meet dietary needs.

Regional disparities further complicate universal adoption. In Ethiopia, for instance, Blakstad et al. [20] found that while plant-based diets deliver lower environmental impacts, the complete substitution of animal proteins may not be feasible for vulnerable groups such as pregnant women and children. Context-specific approaches, including “climate-smart” agricultural practices, are necessary to address these challenges effectively. Additionally, leveraging traditional plant-based cuisines can provide culturally sensitive pathways for dietary shifts, reducing resistance to change.

#### 3.1.3. Synthesis of Perspectives

Considering the evidence reviewed, the environmental implications of PBFs versus meat underscore a complex and context-dependent challenge. While plant-based diets align with broad sustainability objectives, our analysis suggests that their effectiveness depends on regional agricultural practices, supply chain efficiency, processing intensity, and nutritional strategies. From the authors’ perspective, future dietary transitions should not rely on one-size-fits-all solutions. Instead, it is necessary to advocate for a systems-based approach that integrates life cycle thinking, consumer behavior, and socio-cultural factors. Perhaps striking a balance between promoting minimally processed foods and ensuring equitable access to diverse, nutrient-rich options is crucial. Likewise, fostering technological innovations in food processing and enhancing global cooperation on sustainable agricultural practices should be prioritized. Only through such integrative approaches can the shift to PBF achieve its full potential as an environmentally, culturally, and nutritionally inclusive solution. Finally, we acknowledge that in regions facing socioeconomic constraints, supporting sustainable animal agriculture in parallel with biodiversity protection may remain a pragmatic necessity in the short to medium term.

### 3.2. Plant-Based Dietary Pattern Impact on Health

Compared to meat and animal-based foods, PBFs, in addition to the potential environmental benefits not without challenges discussed in the previous section, may offer positive effects on human health beyond their purely nutritional value, and may generate favorable health impacts in a holistic way (human, animal, and environmental well-being) [48]. Consequently, it is relevant to study the human health impact of plant-based dietary patterns compared to those that include animal products.

The methodology for dietary classification was very similar in all studies included. Based on data collected with validated food frequency consumption questionnaires or 24 h dietary recalls, foods were classified into groups, for example, “fruits”, “meats”, and so on. Then a series of points was assigned according to the amount consumed of each food group; for example, the “meats” group was assigned a negative score, while the “fruits” group was positive. Usually, adjustments for caloric intake were also applied in order to equalize all participants. Finally, based on the score obtained, the diet of each participant was classified into a specific dietary pattern. One of the most commonly used ways of classifying dietary patterns is the plant-based index (PDI), which is subdivided into three groups: (1) overall PDI (oPDI), which indicates the general consumption of PBF in the diet (the higher the score, the higher the consumption); (2) healthy PDI (hPDI); and (3) unhealthy PDI (uPDI), which indicate within predominantly plant-based patterns the quality of the foods consumed, generally differentiating between whole plant foods (such as legumes, whole grains, whole fruits, etc.) and other plant foods classified as less healthy, such as fruit juices, refined flours, or potato chips. This methodology has proven to be a robust, validated tool to assess adherence to well-specified dietary patterns in different population groups, both culturally and by age, in population-based studies, and to establish subsequent correlations with health events [49].

Table 1 shows the main characteristics of the studies included, such as the number of participants (*n*), the type of study, the way of collecting food consumption data from the participants, the classification given to the diet of the participants, the years of follow-up, and the age of the study participants. All articles compiled after the literature search are observational prospective cohort studies. These studies allow for the identification of associations between types of diets established for the groups of participants and events measured, whether general mortality (all-cause mortality), inflammation, or cause-specific disease, though establishing causality requires further methodological criteria to establish causal relationships (Bradford Hill criteria). However, observational studies are essential to be able to provide baseline relationships between diet types and health events in a noninvasive and relatively simple and low-cost way.

#### 3.2.1. All-Cause Mortality

In order to establish probabilities of greater or lesser risk of an event (e.g., mortality), the Odds Ratio (OR) or Hazard Ratio (HR) is established based on a comparator. An HR/OR greater than 1 indicates a higher probability of an event, and a ratio less than 1 indicates a lower probability, depending on the comparator established. Regarding mortality, comparisons are made as to whether adherence to a specific type of dietary pattern can increase (HR/OR > 1) or decrease (HR/OR < 1) the risk of all-cause mortality. Table 2 presents the observed risk of all-cause mortality (HR/OR) in each study for each type of dietary pattern.

First, Keaver et al. [50] established a diet quality index, both globally for the diet (cDQI) and for predominantly animal-based (aDQI) and plant-based (pDQI) dietary patterns, as shown in Table 1. A higher intake of healthy foods resulted in a higher index score. The study was conducted with data from the United States (U.S.) population. A comparison was made between those with levels of greater adherence to each of the dietary patterns and those with less adherence, finding an association between greater adherence to aDQI and pDQI and a reduction in the risk of all-cause mortality (HR = 0.75 and HR = 0.66, respectively); however, no significant association was found between aDQI and all-cause mortality, corresponding the HRs shown in Table 2 of the comparison between the highest level of adherence and the lowest level of adherence. Analysis by age group showed a reduced risk (HR = 0.65) in young people (22 to 44 years), with greater adherence to aDQI, whereas in older people (>63 years) this association was observed for pDQI (HR = 0.73), which could be due to the age of the participants. In turn, when discriminating by type of food in terms of risk, it was observed that whole fruits were the group with the greatest risk reduction (HR = 0.72). Finally, a risk reduction (HR = 0.59) was also observed in people with the highest adherence to pDQI and overweight, which was higher than that observed in people with a healthy weight (HR = 0.69). The authors, in turn, highlighted the importance of food quality and its relationship with the events recorded, especially in those plant-based patterns.

On the other hand, Chen et al. [51] catalogued the diet of the participants (data from the Chinese population) with the aforementioned oPDI, hPDI, and uPDI, comparing the highest adherence to each with the lowest. They observed a relationship between a plant pattern (oPDI) and a lower risk of mortality (HR = 0.92), with the risk being even lower (HR = 0.81) when the plant dietary index was healthy (hPDI). However, a higher risk (HR = 1.17) was observed when the index was unhealthy (uPDI). In addition, an HR = 0.86 per 10-unit increase in adherence to hPDI was established, as well as an HR = 1.14 for the same increase in uPDI, although the reduction in risk for oPDI and hPDI, and the increase for uPDI, were not linear. Regarding the food group with the greatest contribution to beneficial association, a risk reduction (HR = 0.78) was observed in people with a daily consumption of fresh vegetables versus those who never consumed them. The inclusion of fish and marine products in the oPDI analysis revealed similar results to the model that did not include them (HR = 0.87).

Similar results were reported by Li et al. [52] using data from the U.S. population and similar methodology (confronting the highest adherence with the lowest, to which the HRs in Table 2 belong), evidencing a lower risk by individuals with higher adherence to oPDI and hPDI (HR = 0.80 and 0.86 respectively), while uPDI was associated with a higher risk (HR = 1.33) in the most adjusted model, which included adjustment for covariates such as race/ethnicity, education level, marital status, poverty ratio, physical activity, smoking, drinking, body mass index (BMI), diabetes, or hypertension, with a linear increase in mortality observed after restricted multivariable cubic spline analysis for greater adherence to uPDI. They also found a non-linear decrease between greater adherence to hPDI and mortality, with this reduction appearing in persons with an hPDI score of higher than 72 (maximum score of 90).

Weston et al. [53] also compared the highest level with the lowest of adherence to each of the dietary patterns (oPDI, hPDI, and uPDI), although the study was conducted with data from the African-American population of Mississippi (U.S.). In the model with the greatest adjustment, which included the covariates of diabetes, hypertension, and total cholesterol, as well as the use of medication for these conditions, education, estimated glomerular filtration, smoking, alcohol intake, physical activity, margarine intake, BMI, age, and sex, a relationship was observed between hPDI with lower risk (HR = 0.94), while uPDI was associated with higher risk (HR = 1.15); however, oPDI was associated with higher risk (HR = 1.07), although these associations were not statistically significant. Surprisingly, an association was found for an additional daily serving of whole grains and a 13% increased risk of mortality. According to the authors, this relationship could be due to a reverse causality or to a low variety in the consumption of this group of foods, and they point out that the results suggest that the benefits associated with PBFs could only be evidenced from a minimum level of consumption, with the quality of these foods being of great importance.

Wang et al. [54], in addition to using the PDI to classify dietary patterns, as presented in Chen et al. [51], Li et al. [52] and Weston et al. [53] also used the PVD—which has more groups for scoring (13 for PDI and 18 for PVD), although it does not include certain foods—as well as the HEI-2015, which represents adherence to the American Dietary Guidelines established by the U.S. government (the higher the adherence, the higher the score), using data from the U.S. population for the study. In addition, participants’ systemic inflammation was measured using C-reactive protein (CRP), which is a biomarker whose increase is related to higher mortality. The highest adherence was compared with the lowest adherence to each dietary pattern. In the model most adjusted for covariates, no association was found between mortality and PDI (both oPDI, uPDI, and hPDI) or with PVD, whereas a lower risk of mortality (HR = 0.87) was observed in those with greater adherence to HEI-2015. A significant association was observed between a severe degree of inflammation (CRP of ≥3 mg/L) and higher mortality (HR = 1.45), although the authors point out the limitation when measuring inflammation and relating it to mortality, since only this marker (CRP) was evaluated.

Zhou et al. [55] also used the PDI to catalogue the dietary pattern of the participants (data from the UK population), comparing the levels of highest adherence with the lowest to each pattern. In the most adjusted model, which included adjustment for the covariates mentioned above and others such as sedentary time, multivitamin supplement use, or menopausal status and hormone replacement use, a reduced risk of mortality was observed in those with higher adherence to oPDI (HR = 0.87) and hPDI (HR = 0.92), while uPDI was associated with an increased risk (HR = 1.29), compared with the lowest adherence. Very strong associations were observed in persons with a higher level of poverty, establishing a 13% lower mean risk in persons with greater adherence to hPDI and a 40% higher mean risk in those with greater adherence to uPDI. The annual mortality rate in the poorest people with the highest adherence to oPDI was 5.22 persons/1000 persons, while it was 5.84/1000 in those with the lowest adherence to oPDI. The authors propose, as explanatory mechanisms for these associations, the lower capacity to acquire quality food products, and point out the importance of promoting equitable access for all people to quality PBFs as a way of reducing mortality among people with lower socioeconomic status.

Yuan et al. [56], in addition to using the PDI used for the classification of dietary patterns by other studies, created a focused index in the population studied (Chinese centenarian population with a mean age of 102.33 years, as seen in Table 1). Thus, based on the dietary characteristics of Chinese older adults, and following the criteria proposed by other scholars in China, they assigned the population, based on the consumption of a series of foods, to the healthy plant-based food index (HPF), unhealthy plant-based food index (uHPF), and animal-based food index (AF). Individuals with higher adherence to each pattern were compared with individuals with lower adherence. In the most covariate-adjusted model, a reduction in the risk of all-cause mortality was observed in those with greater adherence to oPDI (HR = 0.81), hPDI (HR = 0.79), HPF (HR = 0.81), and uHPF (HR = 0.95), although the latter association was not significant, while uPDI and AF were associated with greater risk (HR = 1.10 and 1.17, respectively). Even so, the authors point out certain limitations, especially when it comes to generalizing the results, since in the study, more than 80% of the participants were women, and more than 93% were people of the same ethnicity. However, a whole plant-based dietary pattern and healthy eating practices could reduce the risk of mortality, even in an already relatively healthy population such as centenarians, whose strengthened immune system can lead them to live even longer than 100 years.

Kim et al. [57] performed a study in a multiethnic population (African-American, Japanese, White, Hawaiian, and Latino data population from Los Angeles and Hawaii, U.S.) using the PDI for dietary classification. The highest adherence to each pattern was compared with the lowest. Lower mortality risk was found in women and men with higher adherence to oPDI (HR = 0.89 and 0.85 respectively) and hPDI (HR = 0.86 and 0.88), while uPDI was associated with higher risk (HR = 1.11 and 1.03), as shown in Table 2, although in this case (uPDI) the association was only significant in women. When evaluating each ethnicity separately, a lower mortality risk was found for oPDI and hPDI in all ethnic groups (HR < 1 in all cases), except in Hawaiian women, where no significant association was found (HR = 1.05 for oPDI and 1.00 for hPDI). On the other hand, uPDI was associated with increased risk in both sexes for White participants (HR = 1.13 in men and 1.20 in women), in Japanese women (HR = 1.15), and in Hawaiian women (HR = 1.11). In addition, when analyzing specific dietary components, it was observed that added sugar was associated with an increased risk of mortality, but only in women, and according to the authors, this difference could be explained by hormonal and biological differences between both sexes.

On the other hand, Abris et al. [58] conducted a study in an Adventist population (data from the Seventh-Day Adventist population living in the U.S. and Canada), with the particularity that this population is generally quite health-conscious, so the rate of smoking is relatively low and they regularly practice sports, among other non-dietary factors (covariates) associated with good health. The population was divided into two groups according to the amount of plant-based food consumption (vegetarians and non-vegetarians, although the vegetarian group was subdivided into lacto-ovovegetarians, pescovegetarians, semivegetarians, and vegans), and a reduction in mortality risk (HR = 0.89) was observed for vegetarians compared to non-vegetarians in the model most adjusted for covariates. When comparing the non-vegetarians with the subgroups of the vegetarian category and by sex, it was first observed that male vegans had the lowest mortality risk (HR = 0.72), while in female vegans it was higher (HR = 0.98) than the mean. A lower-than-average risk (HR = 0.82) was noted in male lacto-ovovegetarians, while it was again higher in females (HR = 0.93). As for pescovegetarians, the risk was at the mean (HR = 0.89) in males, while it was slightly higher in females (HR = 0.93). Finally, in semivegetarians, in males the risk was slightly lower than the mean (HR = 0.83), while in females it was higher (HR = 0.98). Even so, in all cases, the HRs observed were <1 compared to non-vegetarians.

Oncina-Cánovas et al. [59] carried out their study with data from the Spanish population, also creating their dietary pattern classification (pro-vegetarian, including overall [oPVG], healthy [hPVG], and unhealthy [uPVG]) with a methodology similar to that established to develop the PDI. Low levels (baseline) were compared with medium and high levels of adherence to each pattern. The HRs shown in Table 2 correspond to the comparison of the highest level of adherence concerning the lowest in the model, with the highest covariate adjustment. A non-significant risk reduction (HR = 0.85) was found in persons with higher adherence to oPVG with respect to those with the lowest, while in those with higher adherence to hPVG, a significant risk reduction was observed (HR = 0.90), which, however, was greater in persons with medium adherence (HR = 0.59). Finally, persons with higher adherence to uPVG were associated with a higher risk of mortality (HR = 1.53), whereas in persons with medium adherence, the risk was lower (HR = 1.31). Even so, the authors point out that the major limitation of the study, as shown in Table 1, is that the study population was very small (n = 597), which could explain the lack of statistical associations for oPVG or the lower risk in persons with medium adherence to hPVG due to limited statistical power.

Huang et al. [60] conducted their study with data from the Chinese population, using the PDI as a classification of dietary patterns. They evaluated the change in adherence to each pattern, establishing the comparison of people with a stable dietary pattern with those who experienced greater change in adherence to a pattern, for both a decrease and an increase. Thus, as shown in Table 2, concerning oPDI, those who increased their adherence the most experienced a non-significant increase in risk (HR = 1.10), while in those who reduced their adherence, the risk was greater (HR = 1.32) and significant. Regarding hPDI, the greatest increase in adherence was associated with a non-significant risk reduction (HR = 0.96), while the greatest reduction in adherence was associated with a significant increase in the risk of mortality (HR = 1.21). Finally, the greatest increase in adherence to uPDI was associated with a higher risk (HR = 1.13), while the greatest reduction in adherence to this dietary pattern was observed to reduce the risk (HR = 0.90) of all-cause mortality. The nonsignificant association between risk and increased adherence to oPDI and hPDI could be due, according to the authors, to the age of the participants (mean 82.2 years, as seen in Table 1), which is higher than in other studies, implying an increase in nutritional requirements, especially in terms of protein content. However, the authors also point out the limitation of not having evaluated the macronutrients of the foods collected in the FFQs, so there is no data to reveal whether deficiencies exist. Even so, it was observed that for each 10-point decrease in oPDI and hPDI, the risk increased significantly by 14% and 21%, respectively, while for uPDI, the risk decreased by 8% in the same case, although this relationship was shown to be non-linear.

#### 3.2.2. Cause-Specific Disease

Regarding specific causes of mortality, the studies by Chen et al. [51], Yuan et al. [56], and Huang et al. [60] only evaluated all-cause mortality. First, for studies with a similar methodology (based on PDI classification), Li et al. [52], in the most adjusted model, found a significant reduction in the risk of cancer mortality (HR = 0.68) in those with greater adherence to oPDI, whereas for hPDI and uPDI, the association was not significant. As for mortality from cardiovascular disease (CVD), the association was only significant in the case of uPDI, with a higher risk (HR = 1.42) found in those with greater adherence to this dietary pattern. On the other hand, in the study by Weston et al. [53], the risk of CVD was evaluated, and in the most adjusted model, a non-significant association was found between oPDI, hPDI, and uPDI and increased cardiovascular risk (HR = 1.09, 1.02, and 1.03, respectively). Furthermore, with regard to specific food groups, a significant risk reduction of 41% was found for each additional daily serving of legumes, while, surprisingly, healthy oils increased the risk of CVD by 10%. However, as in the case of whole grains and the increase in mortality discussed in the previous section, the authors do not rule out reverse causality, which could explain these findings in the case of oils.

In the study developed by Wang et al. [54], an association between PDI and specific diseases could not be established; however, a reduction of between 15 and 21% in CRP was observed in people with greater adherence to hPDI, while the uPDI pattern was associated with greater risk of inflammation (OR = 1.18). In the multi-ethnic study conducted by Kim et al. [57], a lower overall CVD risk was found in both women and men with oPDI (HR = 0.84 and 0.88, respectively) and hPDI (HR = 0.80 and 0.79), while for uPDI the risk was higher (1.08 and 1.19). As for cancer mortality, a significant reduction was only found in men with oPDI (HR = 0.86). When analyzed by ethnicity, a reduction in CVD mortality risk was observed in all ethnic groups for oPDI and hPDI (HR < 1 in all cases), except in Hawaiian women for oPDI (HR = 1. 17), while uPDI was associated with increased risk in both sexes of Japanese (HR = 1.11 men, 1.28 women) and White subjects (HR = 1.19 man, 1.16 women), whereas in African Americans and Hawaiians it was only found in women (HR = 1.19 and 1.39 respectively). For the risk of cancer mortality, no differences were found between ethnicities.

Finally, the study by Zhou et al. [55] evaluated the risk of mortality due to respiratory and neurological diseases, cancer, and CVD. Regarding oPDI, a lower risk of respiratory diseases (HR = 0.85) and CVD (HR = 0.77) was observed, although it was higher for cancer (HR = 1.04) and neurological diseases (HR = 1.03) in those individuals with the highest adherence to this pattern. Higher adherence to hPDI was inversely related to the risk of all pathologies evaluated, including cancer (HR = 0.90), CVD (HR = 0.92), and neurological (HR = 0.65) and respiratory diseases (HR = 0.63), while uPDI was associated with a higher risk of pathologies (HR = 1.16 for cancer, HR = 1.10 for CVD, and HR = 1.54 for respiratory diseases), especially with neurological diseases (HR = 1.95).

On the other hand, in the studies with a more diverse methodology (that did not use or did not only use PDI), Keaver et al. [50] evaluated mortality from cancer and heart disease but found no significant association between these causes of death and any of the patterns used for dietary classification (DQI). However, the study by Wang et al. [54] did find a significant reduction in the risk of CVD (HR = 0.69) and cancer (HR = 0.77) in those with higher adherence to HEI-2015, as well as a reduction in inflammation by 12–15% in those with higher adherence to PVD. In the American Dietary Guidelines, on which HEI-2015 is based, the importance of whole plant foods is emphasized, as well as a progressive reduction in the consumption of animal foods, as discussed below.

In the study conducted by Abris et al. [58] a lower risk was observed in vegetarians than in non-vegetarians for renal failure (HR = 0.52), infectious diseases (HR = 0.57), respiratory diseases (HR = 0.79), diabetes (HR = 0.51), and ischemic heart disease (HR = 0.75); however, in older vegetarians (>85 years), an increased risk of neurological diseases was found (HR = 1.13 for dementia and HR = 1.37 for Parkinson’s disease). The authors hypothesize about the possible relationship between this increased risk and the low, almost null, consumption of polyunsaturated (ω-3) fatty acids (eicosapentaenoic and docosahexaenoic fatty acids, EPA and DHA), although they stress the need for further research in this aspect to better understand this increased risk. On the other hand, when comparing the subgroups of vegetarians with non-vegetarians, the lowest risk was associated with vegans for renal failure (HR = 0.37), pescovegetarians for infectious diseases (HR = 0.41), lacto-ovovegetarians for diabetes (HR = 0.34), and ischemic heart disease for pescovegetarians (HR = 0.61), although for respiratory diseases an analysis by subgroup was not performed. However, an association was also found between dementia and vegans (HR = 1.20) and between Parkinson’s disease and lacto-ovovegetarians (HR = 1.44), which could be due to the reasons mentioned above.

Finally, in the study performed with Spanish population data by Oncina-Cánovas et al. [59], no significant associations were found between oPVG and CVD mortality (HR = 0.99) in persons with greater adherence. On the other hand, a significant risk reduction was observed in persons with greater adherence to hPVG (HR = 0.81), which, again, as was the case with all-cause mortality, was greater in persons with medium adherence (HR = 0.47), which could be due, as stated in the previous section, to the small study population. As for uPVG, a large increase in CVD risk was observed in those with the highest adherence (HR = 2.10).

Therefore, a greater adherence to a plant-based dietary pattern, and especially to a healthy one, could be associated with lower all-cause as well as specific-cause mortality, as observed in the results of the studies considered in this section, although the importance of food quality should be emphasized, since while plant-based whole foods would be associated with better health compared to a predominantly animal-based pattern, unhealthy plant-based foods are also associated with worse health outcomes, as shown by the increased risk of uPDI. Notwithstanding the associations observed in these studies, the vast majority of authors point out limitations in methodology. The main limitation of these studies is the form of data collection (FFQ or 24 h diet recall), since consumption is established subjectively by the participants and, in some cases, does not record dietary changes during the study time, and although these questionnaires are pre-validated, they can be misleading. Second, as mentioned above, survivor bias or reverse causality may occur when establishing an association between a dietary pattern and mortality. Finally, in no case can causality be inferred from the included studies alone.

In the review conducted by Neufingerl & Eilander [61], it was observed that people with a plant-based dietary pattern presented a higher risk of deficiency of vitamins B_12_ and D, ω-3 polyunsaturated fatty acids (EPA and DHA), and certain minerals, especially in those with a 100% plant-based diet (vegans) compared to meat-eaters, although the latter presented a higher risk of deficiency of fiber, vitamins B_9_ and E, polyunsaturated fatty acids, and magnesium. Even so, the trend observed in the included studies is that a plant-based diet composed predominantly of whole foods is associated with a reduced risk of overall mortality, specific pathologies such as cancer or cardiovascular disease, and indicators of inflammation. Despite the above-mentioned limitations, the observed results correlate with dietary recommendations established by the main health organizations worldwide, such as the Dietary Guidelines for Americans 2020–2025 (the pre-2020 version of which the HEI-2015 pattern is based on), established by the United States Department of Health and Human Services (USDHHS) [62], or the Dietary Recommendations for the Spanish population set by the Spanish Agency for Food Safety and Nutrition (AESAN) [63], where it is recommended to reduce cholesterol intake and keep saturated fat intake to <10% of total energy intake and trans fat intake to <1% of total energy intake, with these compounds coming mainly or exclusively from foods of animal origin. In addition, it is also recommended to maintain the intake of free sugars at <10% of total energy intake and a salt intake of less than 5 g per day. The origin of these compounds generally comes from foods classified as unhealthy, of both plant and animal origin.

Thus, in the study by Cutroneo et al. [64], which compared the nutritional composition of meat analogues (MAs) to their meat counterparts, it was observed that in MSs compared to real meat products, the average energy was higher, related to their higher total lipid content. However, the fat profile was different. In all types of MSs analyzed (steaks, burgers, meatballs), the saturated fat content was lower than in meat controls. As for carbohydrate content, it was observed to be higher in terms of total and fiber content in all analogues, although the free sugar content was also higher than in meat controls. Protein content was similar to that of the meat controls except in the burger and meatball analogues, where it was observed to be slightly lower; however, the amino acid profile was observed to be different from that of meat, depending on the raw material used to formulate each MS. Finally, sodium content was similar to that of the meat products, except in the ready-sliced MAs, where it was lower, with the highest salt content being found in the cured meat. In terms of ingredients, the number was higher in the analogues. Thus, although the sodium and sugar content is similar or higher than that of meat products, MAs have a greater amount of fiber; a similar protein content, although with a different amino acid composition; and a better lipid profile, so the substitution of meat for its analogues could better fit in with the recommendations given by official organizations, although research and development of analogues with lower sodium and sugar content should continue.

These organizations (USDHHS, AESAN) also emphasize the importance of consuming a majority of whole PBFs in the diet as a way to maintain good health within a healthy lifestyle framework that includes regular exercise and the absence of consumption of toxic substances such as tobacco or alcoholic beverages. At the same time, the importance of promoting a regulatory environment that favors the easy and cheap acquisition of whole vegetal foods by the population should be emphasized, with the possibility of reducing taxes on healthy foods and/or increasing taxes on others that may pose a greater health risk compared to the latter, together with public campaigns to promote healthy eating and lifestyles. Thus, based on the evidence and in accordance with the recommendations of health organizations, it can be deduced that a plant-based dietary pattern composed mainly of whole-grain foods could be associated with health benefits beyond their nutritional value, such as a reduction in the risk of all-cause and cause-specific mortality, as well as a lower environmental impact, as described before, and that novel PBFs fortified with certain nutrients such as vitamin B_12_ should be included to prevent possible micronutrient deficiencies, with as little salt and as few free sugars as possible.

### 3.3. Innovative Ingredients and Their Environmental Impact: Feasibility of Future Meat Alternatives

Considering the benefits of PBFs compared to meat and animal-based diets, alongside the existing challenges and limitations of fully transitioning to a plant-based food system, there is a growing interest in identifying alternative protein sources, whether plant- or animal-derived, that offer a lower environmental impact. In this context, novel foods are emerging as promising alternative protein sources, with insects, cultured meat, mycoprotein, and microalgae emerging as potential protein alternatives, although their environmental and nutritional benefits are still subject to ongoing evaluation and debate. These innovative protein sources broaden the spectrum of meat alternatives by employing novel production technologies that may overcome specific scalability, nutritional completeness, and consumer acceptance challenges faced by conventional plant-based foods. However, similar to plant-based foods, critical aspects remain associated with these novel protein sources, particularly regarding their production systems, economic viability, and sensory acceptance [25], which will be addressed throughout the subsequent sections of this study.

#### 3.3.1. Edible Insects

Edible insects have been traditionally consumed in various parts of the world, mainly in Asia and America. In recent years, their consumption has been introduced in the European Union. Since 2021, some insect species have been included as novel foods in the European Union (mealworm, migratory locust, domestic cricket, and dung beetle), mainly in their dried, ground, and frozen form [65,66,67,68,69,70].

Several studies discuss the environmental impact of edible insects. These foodstuffs have been proposed as a more environmentally friendly protein source alternative to meat. Vinci et al. [71] conducted LCA, concluding that mealworm production requires fewer fossil resources and land use, and implies fewer climate-altering emissions than pork. Dreyer et al. [72] performed a similar study but compared it with broiler production. LCA indicates that mealworm production entails a lower environmental impact in all five categories studied (kg CO_2_-eq of global warming potential, MJ-eq of non-renewable energy use, m^2^ of agricultural land occupation, g SO_2_-eq of terrestrial acidification potential, and g P-eq of freshwater eutrophication potential). The differences ranged from 18 to 72% depending on the impact category studied. Edible insect production is also characterized by a more efficient resource use, as they have a high food conversion ratio to produce the same amount of protein, e.g., crickets require only one-sixth of the feed needed for cattle, one-fourth of that required for sheep, and half the amount consumed by pigs and broiler chickens [73,74]. Accordingly, all the authors agree that edible insects represent a sustainable meat alternative for human consumption [71,72,73,74,75,76,77,78,79,80,81]. Furthermore, Spykman et al. [79] performed a holistic approach between environmental impact (LCA) and economic feasibility (life cycle costing) called eco-efficiency assessment, and describe the production of black soldier fly larvae as not only environmentally favorable but also economically viable.

However, the sensory aspect is still a challenge in terms of insect consumption, especially in Western society, where consumers tend to reject edible insects. In this regard, recent studies suggest insect enrichment of commonly consumed foods for greater consumer acceptability so that the insect is not visibly present in food [73,81,82]. Some cricket-enriched foods that have shown technological and sensory quality are pasta, bread, beef burgers, sausage patties, muffins, and brownies [78,83,84,85]. This may be a useful strategy to adapt the Western population to the consumption of edible insects. Even with only 5 to 10% enrichment, these fortified foods still have an environmental benefit, as shown by Smetana et al. [78] when performing an LCA comparing an insect-enriched beef burger versus the non-enriched burger. In addition, a great consumer acceptability was obtained for the enriched burger.

Considering the environmental and economic suitability of insect production, and at the same time the challenge of their consumer acceptance, the introduction of edible insects in other links of the food chain that do not involve direct human consumption may be of interest. In this sense, their application in livestock [79,86,87,88] and fish [89,90] feed, as well as their usefulness for the bioconversion of livestock waste for application as agricultural fertilizers [91,92,93], have been studied in recent years. Both applications have shown promising results, not compromising animal or agricultural production yields, and representing an alternative to commonly used fertilizers and animal feeds, which are often more environmentally friendly.

On the other hand, insects possess high nutritional quality, which should be considered when evaluating the suitability of meat alternatives. Edible insects are characterized by their mineral content (especially P, K, Ca, Mg, and Zn), lipid profile (higher proportion of unsaturated to saturated fatty acids), and high protein quality. Cricket, for instance, exhibits a rich profile of essential amino acids, constituting approximately 42.7% of its total amino acid content. In particular, it contains significant amounts of valine (4.5 g/100 g), leucine (3.8 g/100 g), and isoleucine (2.9 g/100 g). These levels are comparable to those found in traditional animal protein sources such as eggs, pork, chicken, and beef. A comparison between several insect species and meats indicates that both are sources of complete animal protein, containing all essential amino acids in their composition. In addition, edible insects have a remarkable fiber content (2.1–22.2 g/100 g) depending on the insect species [94,95]. The main source of fiber in insects comes from the chitin of their exoskeleton. Evidence of the effect of chitin on the organism is scarce; however, a possible bifidogenic effect on the gut microbiota has been shown in healthy subjects after regular consumption of a cricket-enriched meal [96].

#### 3.3.2. Cultured Meat

Cultured meat is one of the most studied meat alternatives in recent years. Cultured meat differs from MAs because its production involves an in vitro cell culture or genetically modified organisms [97]. Environmentally, cultured meats have been described to have a lower impact than conventional meat, especially compared with beef [77,78,98,99,100]. Cultured meat implies lower use of land, water, and energy, as well as fewer GHG emissions based on LCAs. However, some authors assure that relevant factors are often not taken into account. Hu et al. [101] claim that regarding LCA of cultured meat, existing studies lack practical data at a commercial scale. Indeed, some studies recognize as a limitation that LCA is based on small-scale production [77]. As described by Soccol et al. [99], technological and economic challenges must be overcome to make large-scale cultivated meat production a real perspective.

In this sense, Risner et al. [102] and Wali et al. [103] agree to focus on the development of an optimum cell-growth medium that allows for the scale-up and economic feasibility of cultured meat. This has been described as one of the main limitations of cultured meat production, as the cell-growth media currently used (which contain ingredients of animal origin such as fetal bovine serum) are responsible for cultured meat having a potentially greater environmental impact than beef [102]. Other limitations linked to the commercial scale could be the excessive demand for critical materials or the significant amounts of energy required in the production process. In this regard, Noble et al. [100] indicate that significant reductions in GHG emissions are expected compared to traditional beef farming, but for other meats like pork and chicken, the potential GHG reductions from cultured meat are largely influenced by the incorporation of renewable energy sources in the production process. Accordingly, an ex-ante LCA of commercial-scale production agrees that energy use is one of the main drawbacks, with the energy source being a determining factor for the environmental impact of cultured meat [104]. Tuomisto et al. [105] demonstrate that greater technological advancements (culture media, energy efficiency and sources, scalability) are necessary for the environmental impact of cultured meat to be lower than that of conventional meat. Other studies agree that cultured meat has great potential for improvement to become a more sustainable meat alternative, but it remains a challenge [80,106].

On the other hand, consumer acceptance of cultivated meat is moderate but steadily increasing, influenced by various demographic and regional factors. Studies indicate significant differences across geographic areas and population groups. Consumers who prioritize environmental sustainability and animal welfare tend to be more receptive to cultured meat. However, major barriers persist, particularly regarding perceptions of naturalness, safety concerns, and the so-called “disgust factor”, which continue to hinder widespread adoption. Addressing these concerns through transparent communication and consumer education will be essential for fostering greater acceptance [107].

Regarding the nutritional value of cultured meat, it has the potential to replicate the composition of farmed meat. However, challenges persist in imitating the amino acid composition, fat profile, and micronutrient supply. Improving growth media, scaffold materials, and fortification strategies will be crucial to enhancing the nutritional quality. Further studies are required to ensure that cultured meat meets dietary needs and consumer expectations while adhering to food safety and regulatory standards [108].

#### 3.3.3. Mycoprotein

Mycoprotein is a foodstuff obtained from the fungal fermentation of carbohydrate-rich substrates, followed by the extraction and processing of the resulting biomass [109]. The fungal biomass contains approximately 45% protein (dry weight) and exhibits a fibrous structure that closely resembles muscle tissue, making it particularly suitable for the development of MAs with textures comparable to conventional animal-derived products [25]. Currently, *Fusarium venenatum* is the main fungal species from which mycoprotein is obtained [110], although species from other genera, such as *Aspergillus oryzae*, are also being studied [111]. The metabolic versatility of filamentous fungi enables them to utilize diverse carbon sources, including agricultural residues and industrial by-products. This characteristic presents significant opportunities for the food industry to make use of by-products while simultaneously reducing environmental impacts through circular economy principles [109].

Several studies indicate that mycoprotein has significantly lower environmental impacts than meat [65,66,67,68,69,70]. GHG emissions from mycoprotein constitute less than 14% of the emissions associated with beef protein production [109]. Direct comparisons between mycoprotein burgers and beef ones show that this MA generates an impact six to seven times lower, with lower GHG emissions (1.62–1.8 kg CO_2_eq/kg vs. 9.04–9.15 kg CO_2_eq/kg), land use (3.3–3.6 m^2^/kg vs. 10.94–10.96 m^2^/kg), freshwater eutrophication, and terrestrial acidification [78].

Environmental critical point analysis reveals that the main contributor to the impacts of mycoprotein is energy consumption during fermentation and processing, accounting for approximately 70% of the total environmental impact [80]. In this sense, the sustainability of mycoprotein could be significantly improved by integrating renewable energy sources into the production system [25]. The notable advantage of mycoprotein is its minimal dependence on arable land compared to animal- and plant-based proteins, especially when using food by-products as the fungal fermentation substrate [112]. Glucose derived from the starch of food crops, mainly maize or wheat, is the substrate conventionally used to obtain mycoprotein. Alternative substrates and by-products such as rice straw, miscanthus, and switchgrass imply a lower environmental impact than conventional ones in mycoprotein production [109].

Economically, mycoprotein production remains considerably more expensive than conventional meat. However, commercial mycoprotein-based MAs such as Quorn are already available on the market and have demonstrated good sensory acceptance among consumers [78]. Techno-economic analyses suggest that production costs could be significantly reduced through process optimization, particularly by utilizing food industry by-products as substrates for fungal fermentation [109].

From a nutritional standpoint, mycoprotein and its derived MA exhibit a highly favorable profile, which may contribute to consumers’ willingness to pay a premium. Mycoprotein provides a complete amino acid profile with high digestibility, resulting in a protein quality comparable to that of meat and superior to most plant-based proteins [113]. Its lipid composition is characterized by a low total fat content, a reduced proportion of saturated fatty acids, and a predominance of polyunsaturated fatty acids, including ω-3s, with a favorable ω-3/ω-6 ratio (1:6). Furthermore, mycoprotein contains a substantial amount of dietary fiber (25% dry weight), primarily composed of β-glucans (two-thirds) and chitin (one-third) [114].

Altogether, mycoprotein represents not only a more sustainable alternative to meat but also a nutritionally advantageous one.

#### 3.3.4. Microalgae

Microalgae are photosynthetic microorganisms with the potential to meet sustainable food supply needs, particularly regarding protein demand. Although the number of microalgae species in nature is estimated to be between 200,000 and 800,000, only a few are used in food applications, mainly species such as *Nostoc*, *Arthrospira* (commonly marketed as spirulina), *Aphanizomenon*, *Chlorella*, *Dunaliella*, and *Haematococcus*. These microalgae are incorporated into various food formats, including supplements (pills, capsules), extracts (β-carotene, phycocyanin, DHA), and ingredients in food products such as emulsions, gels, dairy products (yogurts, cheeses), cookies, bread, and pasta, enhancing their nutritional profile and providing potential health benefits through bioactive compounds [115].

Scientific literature demonstrates a wide range of applications for microalgae with environmental benefits. In recent years, research has focused on using microalgae in biorefinery, representing a sustainable alternative for the production of biofuel, energy, and chemicals of interest. In this context, researchers aim to leverage microalgae’s capacity to metabolize CO_2_ and other GHGs, thereby reducing their atmospheric concentration while obtaining valuable compounds from the created biomass [116].

In the food sector, microalgae utilization is diverse and extends beyond direct human consumption. One of the primary applications is waste management. Their use in the reutilization of food byproducts has been described and optimized [117,118,119,120,121,122,123]. Similarly, several studies investigate their application in wastewater treatment [124,125,126], specifically from the wine industry [127], olive oil production [128], and even cow manure [129]. The use of wastewater and food waste for microalgae cultivation has also been studied for obtaining bioproducts with medicinal value [130] or pigments [125]. They can also serve as biofertilizers [125,131], including through the recovery of nitrogen from agri-food waste for subsequent use as biofertilizers [132]. Another interesting application is their use as bioplastics [133], which can also be produced from recycled organic compounds [122]. In summary, all aforementioned studies attempt to prevent waste disposal into the environment by utilizing these residues for microalgae growth. Subsequently, the obtained biomass has multiple applications that can derive products from waste and thus promote circular economic development.

Regarding production for human consumption, microalgae have been studied as one of the sustainable protein alternatives to meat [75]. Their favorable environmental impact has been reported [134,135,136]. Specifically, the environmental impact of producing 1 kg of protein from microalgae (*Chlorella vulgaris*) is substantially lower than that of animal sources across multiple indicators. Microalgae production results in up to 88% less global warming potential, 87% less land use, and 83% less scarcity-weighted water use compared to beef production. Specifically, meals containing microalgae showed significant reductions in environmental impacts: 95% less freshwater eutrophication, 78% less marine eutrophication, and 92% less terrestrial acidification compared to animal-source meals. When environmental impacts are integrated with nutrient richness in nutritionally invested environmental impact indices, microalgae rank substantially better than beef, chicken, fish, and pork, offering nutritionally equivalent protein with a dramatically reduced ecological footprint. This positions microalgae as a promising sustainable protein alternative for future food systems. In addition, microalgae contain polyunsaturated fatty acids, carotenoids, fiber, vitamins, and minerals lacking in conventional meat [25].

However, their production process still presents several environmental limitations. Microalgae are primarily commercialized in dry format, and the drying process significantly increases the environmental impact of production, partly due to energy consumption [137]. Additionally, they use CO_2_ for growth, which is manufactured in ways that increase environmental impact, as direct use of atmospheric CO_2_ is still being optimized. An interesting alternative that has shown a favorable environmental impact is the use of CO_2_ from the brewing industry [138].

Furthermore, beyond production for direct human consumption, some studies describe the favorable environmental impact of using microalgae for animal feed compared to commonly used feeds, especially in aquaculture [131,139,140,141].

In conclusion, microalgae for human consumption constitute a quality protein source and a nutritionally favorable alternative to meat, with demonstrated lower environmental impact. However, various challenges remain in optimizing microalgae production to improve yield and environmental impact, so further research is needed in this field. Additionally, microalgae provide enormous potential for managing food industry waste to create compounds of interest both in the food sector and many others, thus contributing to an additional environmental benefit.

#### 3.3.5. Comparison of Meat-Alternative Foodstuffs

Conventional and innovative meat-alternative protein sources and their environmental impacts have been extensively examined. Based on the evidence reviewed in this study, these alternatives demonstrate significant potential to reduce environmental burdens compared to conventional animal proteins. In particular, several studies provide detailed comparisons in this regard, as summarized in Table 3 [18,25,37,47]. A prominent disparity is evident between conventional meat and PBFs concerning environmental impacts. For instance, beef production results in substantially higher GHGs, land usage, and water consumption compared to plant-based alternatives like lentils and soybeans. Similarly, novel foods, including cultured meat, insects, mycoprotein, and microalgae, represent promising alternatives to conventional animal proteins, offering reduced environmental impacts while maintaining comparable or superior nutritional profiles characterized by complete amino acid compositions, high micronutrient density, favorable lipid profiles, and significant fiber content. Nevertheless, significant challenges remain in the production of novel foods that must be addressed to reduce associated environmental impacts and economic costs [25], which are identified below.

Synthesizing the key limitations identified throughout the preceding sections, several critical constraints emerge across novel protein production systems. For instance, edible insects require controlled environmental conditions with associated energy costs, while feed composition influences sustainability metrics. Cultured meat production is limited by high energy demands, reliance on expensive animal-derived growth media, and critical material requirements. In addition, current assessments lack commercial-scale data, and benefits depend heavily on renewable energy integration. Mycoprotein’s environmental burden stems primarily from fermentation and processing energy consumption (approximately 70%), with conventional systems utilizing food crop-derived glucose. Microalgae production faces constraints from energy-intensive drying processes and manufactured CO_2_ inputs, as direct atmospheric CO_2_ utilization remains suboptimal.

Notwithstanding these production challenges, comprehensive LCAs establish a clear environmental hierarchy among alternative protein sources. Insects emerge as the most sustainable option, consistently ranking the lowest across multiple impact categories [8,77,113] due to exceptional feed conversion efficiency, minimal land and water requirements, and high edible fractions. Microalgae demonstrate variable performance contingent upon cultivation systems, with heterotrophic methods yielding superior outcomes compared to autotrophic photobioreactor systems [77,80], while maintaining consistent land use advantages. Mycoprotein occupies an intermediate position with moderate impacts driven by fermentation energy demands, despite controlled production environments offering optimization potential [77,80,113]. Cultured meat consistently exhibits the highest environmental burdens due to intensive energy requirements and nascent technological development [77,80,113]. In short, energy consumption emerges as the primary determinant of environmental performance across all alternative protein sources. This convergence suggests that renewable energy integration represents the most effective strategy for enhancing sustainability, particularly for energy-intensive systems, including cultured meat, mycoprotein, and autotrophic microalgae cultivation platforms.

**Figure 2:**
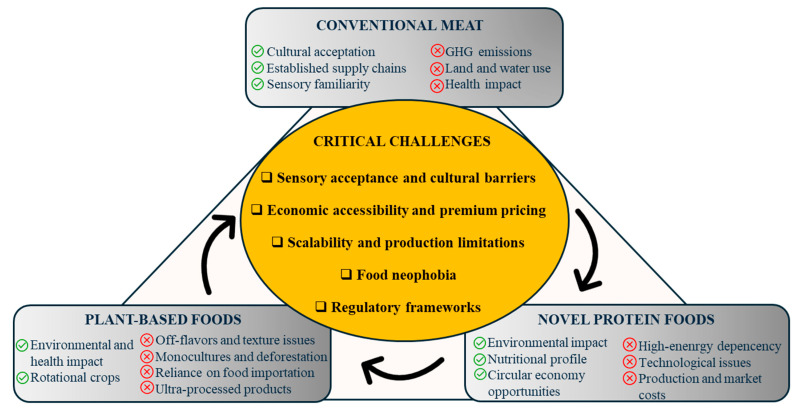
Comparative framework of protein sources: advantages, limitations, and critical implementation challenges for sustainable protein transition.

At this juncture, Figure 2 is presented to visually synthesize the key aspects of conventional meat, as well as of the conventional and novel alternative protein foods discussed thus far. This conceptual framework illustrates the comparative advantages and limitations of each protein category while highlighting the critical implementation challenges that transcend individual protein sources. These multifaceted barriers will be further explored throughout the subsequent sections.

### 3.4. Consumer Sensory Acceptance Analysis of MS

Plant-based MSs and innovative protein foods could be more environmentally sustainable and have a better health impact than their MAs, but this may be futile if consumers finally decide to choose conventional meat or animal products over these alternatives. Consumer preference for certain products depends on a wide variety of factors, like economic, social, or psychological factors, but the sensory characteristics of the product are an important aspect when purchasing them.

#### 3.4.1. Sensory Perception

The sensory acceptability of a product can be evaluated by hedonic scales, where various parameters (flavor, texture, appearance, smell, etc.) are evaluated with a point system. In addition, specific properties such as texture or aroma can be subsequently evaluated by means of other questionnaires that include specific aspects for these items.

Thus, Smetana et al. [78] evaluated the acceptability of plant-based burgers marketed in Germany, where a comparison was made between plant-based burgers made with soy, insect, mycoprotein, and pea and a standard meat burger, which acted as a reference (control). A hedonic test was performed to determine the general acceptance of the burgers, and then specific parameters such as meat taste or color were evaluated, and finally, the participants (*n* = 126) were asked whether they would buy the burgers and how much they would be willing to pay for each one. The burger with the best overall rating in the hedonic analysis was the insect burger, followed closely by the meat burger, while the worst rated was the soy burger. As for the analysis of specific parameters, the soy-based burger was rated as the burger with the best bite firmness and the pea-based burger as the best in terms of color, while the beef burger was rated as having the best flavor and also the best meat taste, with the salt taste rating being the same for beef and insect burgers. Regarding the willingness to buy and how much to pay for the burgers, participants expressed that they would be willing to buy the beef burger, followed very closely by the insect burger, while they would not buy the soy burger. They also expressed that the highest price they would pay would be for the insect burger, followed by the meat burger, while again, they would pay the lowest price for the soy burger [78]. It is therefore observed in this study that some vegetable alternatives, especially the insect-based ones, are well evaluated and comparable to meat; however, it is worth mentioning that it depends on the commercial brand, as well as certain limitations of the study, since it did not take into account the evaluation of psychological aspects of the participants, such as food neophobia, which also influence food acceptance.

On the other hand, Niimi et al. [142] evaluated the acceptability of five Bolognese sauces made with MSs (3 soy-based, 1 oat-based, and 1 mycoprotein-based), compared with one made with minced meat. The participants who analyzed the food (*n* = 101), in addition to performing hedonic scales on the sauces, filled out questionnaires on food neophobia and frequency of food consumption. The sauce with the highest liking was the one made with a meat alternative soy-based sauce, followed by meat, and the one with the lowest liking was oat-based, and the soy-based sauce was the one with the highest acceptance in terms of appearance, aroma, flavor, and texture, with significant differences in appearance compared to all the other sauces. The meat-based sauce came second in all parameters except appearance, and the oat-based sauce was always the worst-rated. In terms of psychological aspects, food neophobia was related to a lower predisposition to liking in terms of appearance, as well as overall liking and texture. People with lower food neophobia, greater exposure to meat alternatives, and greater culinary skills expressed greater sensory acceptance of MSs, although the type of product and vegetable protein used was seen to be important in terms of acceptance.

Thus, a large proportion of MSs are still worse valued sensorially than meat today, although certain brands of vegetable alternatives can already be compared or are valued as even better. However, the studies included to evaluate the acceptance of these products may not reflect the global consumer pool, as they were conducted in Western populations, while in other populations and cultures, the acceptance of specific alternatives, such as insects, is more accepted since they are consumed traditionally.

#### 3.4.2. Factors Affecting Acceptance

Sensory acceptance is important when choosing a meat alternative, and some products are already sensorially more highly rated than meat. However, there is a variety of factors that influence this acceptance. For people who do not consume meat alternatives, intrinsic characteristics of the product, such as texture, visual appearance, or taste, seem to be one of the most important aspects influencing them to start consuming them, together with food neophobia and low information about the product [143], in line with the results observed by Niimi et al. [142], with older people, those with low education, and those living in small cities being the ones who generally accept these products less [144]. On the other hand, environmental and health concerns are related to higher acceptance, especially if information about these aspects of the product is provided, although price is also an important aspect in the choice of these products, even if they are sensorially accepted.

For specific novel foods, Laureati et al. [145] have found that acceptance for aquatic foods, such as microalgae, is strongly affected by food neophobia and sensory aspects, while perceived healthiness and product knowledge reinforce acceptance. A culinary culture open to new experiences also seems to have a positive impact on the acceptance of these products, with observations in this study of how in France, whose culinary tradition is held in high value and, therefore, where changes and new foods are generally not well accepted, this type of product is much less accepted than in neighboring countries such as Germany or the Netherlands. As for insects, food neophobia and appearance aversion strongly influence the rejection of insects, although less so in young people, whereas insect-derived products, where insects are not seen, are better accepted, in agreement with the results observed by Smetana et al. [78], where the insect-derived burger was well accepted. Again, the cultural factor is important, since it has been observed that in Asian countries, where the consumption of insects is more widespread than in European countries, the acceptance is higher, both for processed products (e.g., insect meal) and for ready-to-eat insects [145]. Regarding cultured meat, the belief that it is not a natural product is the main psychological barrier for consumers. Other socio-cultural factors, such as certain religious issues linked to the use of cells during the production process, lead to the rejection of these products. In addition, although the appearance of cultured meat is usually similar to that of meat, the taste generally differs, which is another difficulty facing its acceptance. One of the great difficulties facing the acceptance of these products is the socio-economic conditions, since their development still requires considerable resources, and therefore, the cost is usually high [146]. Finally, plant-based products are better accepted by more educated people and if they are perceived as healthier and more sustainable, although again, food neophobia decreases their acceptance.

**Figure 3:**
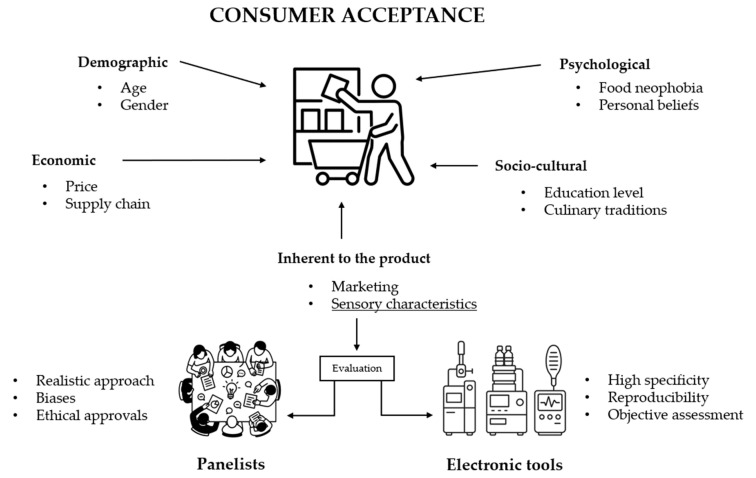
Factors influencing consumer acceptance and forms of sensory evaluation of meat analogues.

Therefore, consumer acceptance depends on many interrelated factors, as shown in Figure 3, including economic (prices and supply chain), psychological (food neophobia, personal beliefs about the naturalness of a product, chemophobia), socio-cultural (level of studies and education, religious beliefs, regional food systems, culinary traditions), and demographic (age, gender), as well as on the product itself (sensory aspects, marketing, consumer familiarity) [147]. Therefore, there is no single way to improve the acceptability of these products, and a multi-pronged approach is needed. Increased supply of these products could reduce food neophobia by increasing their familiarity to consumers while reducing the price by expanding supply, making them more affordable for the lower-income segments of the population. In turn, advertising campaigns designed for consumers with a lower acceptability of specific alternative plant proteins could change their behavior, while educational awareness campaigns at the population level could reduce the reluctance of older segments of the population to acquire them. At the policy level, the production of existing meat alternatives could be encouraged. On the part of the food industry, the incorporation of hybrid products, initially substituting a small amount of meat for its plant-based alternatives, and increasing this substitution over time, could also improve acceptability.

On the other side, although meat tends to be currently valued as equal or superior in sensory aspects, there is a growing acceptance of meat alternatives, partly due to innovation and improvements made in recent years by the food industry, especially among young people, as they tend to be more environmentally conscious, and it should be noted that continued increasing exposure to these new foods (for example, in public canteens) could improve their acceptance, although the sensory aspects of MAs must continue to be improved in order to get more products on the market with meat-like characteristics. Sensory improvements can be evaluated not only through evaluations with people, who may have some biases and which would require ethical approvals, but also through electronic evaluation, an emerging novel alternative to reducing these potential problems.

### 3.5. Analytical Evaluation of MSs and PBFs Through Electronic Sensors

Sensory assessment of food has traditionally been carried out through human panelists. Economic costs, ethical considerations, and psychological biases are some of the disadvantages associated with human panelists, something that was slightly discussed in the previous section. In this sense, electronic sensory technologies (e-panel) (comprising an e-eye, e-nose, and e-tongue) mimic human sensory perception and offer a rapid, automated, and unbiased alternative compared with conventional methods [148], as summarized in Figure 3. Currently, efforts are focusing on the development of PBFs, protein meat alternatives, and MAs. In this context, e-panels have been demonstrated to be a valuable resource for the sensorial evaluation of these products. Several studies have employed electronic panel (e-panel) techniques to optimize the organoleptic properties of MAs and other PBFs, such as plant-based beverages.

#### 3.5.1. MA Electronic Sensory Analysis

Regarding MAs, several studies, like Keum et al. [149], focused on the development of plant-based patties incorporating hydrogel beads as flavor retention agents. In this study, an e-nose was employed to optimize the patties’ flavor by selecting the hydrogel beads that best preserved desirable aroma characteristics during storage (sodium alginate/β-cyclodextrin hydrogel beads). Furthermore, the results obtained with the e-nose were consistent with those from a trained sensory panel, highlighting the potential of this technique as a valuable tool in the formulation of MAs. Similarly, an e-nose was employed to evaluate a plant-based beef analogue, revealing that the incorporation of *Pleurotus ostreatus* mycelium enhanced the meat-like aroma. This helped improve the overall flavor profile of the product [150]. In this study, an e-tongue was also employed, and it suggested that the mycelium supplementation may decrease bitterness and astringency, which was in agreement with the sensory evaluation conducted by trained human panelists. In line with this, the incorporation of *Haematococcus pluvialis* into a plant-based MA has been shown to enhance its flavor profile, particularly by imparting grassy and herbal grain-like notes, as evaluated through e-nose technology. Moreover, this microalga exhibits strong coloring capabilities, primarily attributed to its high astaxanthin content, which contributes to a red hue closely resembling that of conventional red meat [151].

Another study compared three commercial MAs with beef patties, two based on soy protein and one on pea protein. E-nose analysis identified organic sulfur compounds and nitrogen oxides as the main aroma contributors in both beef and plant-based samples. Strikingly, the pea protein-based patty exhibited the highest aroma stability after thermal processing and the closest aroma profile to that of cooked beef. However, all three plant-based patties lacked a fat-like aroma and presented an excessively strong spice aroma. E-tongue analysis further indicated that plant-based patties tended to display higher bitterness and astringency compared to beef. Interestingly, PCA results revealed that the MAs were not more similar to each other than to beef, suggesting fundamental differences in the flavor profiles of both meat and its analogues. The findings from the e-nose and the e-tongue were complementary and aligned with those obtained from GC-MS and human sensory panels, supporting the effectiveness of e-panel technologies for the sensory evaluation of plant-based products [152].

Bakhsh et al. [153] employed e-tongue technology to assess the effect of red yeast and lactoferrin supplementation on the taste profile of plant-based patties. The plant-based formulation exhibited increased umami perception, while a negative trend in acidity was also observed. The authors attributed the elevated acidity to the presence of tannins derived from vegetable proteins and added spices commonly used in MAs. Additionally, the incorporation of red yeast and lactoferrin significantly enhanced the color, achieving a closer resemblance to conventional red meat, and improved key textural attributes such as gumminess and cohesiveness. These findings suggest that both red yeast and lactoferrin hold promise as functional ingredients for the sensory enhancement of MA products. In another study, e-tongue analysis demonstrated that quinoa flour-based patties exhibited a sensory profile more closely resembling that of conventional meat compared to rice flour-based counterparts. Nonetheless, the plant-based patties showed higher levels of astringency, bitterness, and saltiness, while umami and acidity perceptions were comparable to those of beef patties [111].

Despite promising advances in MAs, significant sensory challenges persist in achieving optimal consumer acceptance. Partial substitution of animal protein with plant-based proteins, as briefly outlined at the end of the preceding section, represents a strategic approach that may facilitate the transition toward increased consumption of MAs. This hybrid formulation strategy effectively mitigates the negative sensory attributes frequently associated with complete meat replacements while maintaining desirable organoleptic properties [154]. In this sense, Bakhsh et al. [155] evaluated the sensory profile of beef hamburgers formulated with textured soy protein at substitution levels ranging from 10% to 40%. At the highest level of substitution (40%), certain sensory attributes were affected: Astringency increased significantly, while richness decreased. Interestingly, saltiness showed a progressive increase with higher substitution levels, whereas bitterness decreased. Furthermore, the 40% substitution level yielded favorable scores for acidity and umami, suggesting an overall promising sensory profile for meat products enriched with soy protein at varying concentrations. Correlation analysis identified significant relationships between specific fatty acid composition and sensory attributes: Myristoleic acid (C14:1) positively correlated with bitterness perception and linoleic acid (C18:2) showed a positive association with saltiness, while palmitic (C16:0) and stearic (C18:0) acids exhibited negative correlations with astringency. These findings provide valuable insights for optimizing hybrid meat product formulations that balance nutritional enhancement through plant protein incorporation while maintaining sensory appeal, potentially offering an effective pathway toward more sustainable dietary patterns without compromising consumer acceptance.

#### 3.5.2. Other PBF Electronic Sensory Analysis

In addition to MAs, plant-based beverages are the main PBF in which electronic sensory analysis has been applied, so the following is a description of the studies that apply these new technologies to plant-based beverages. Thus, in the study by Demeter et al. [156], plant-based milks (soy, hazelnut, rice, coconut, and almond) were compared with milk (normal [3.5% fat] and semi-skimmed [1.5% fat]) to produce freeze-dried milks to serve as drug carriers, since milk has traditionally been used. As part of the study, which of the plant-based milks had the most similar profiles to milk was investigated using an electronic tongue system (*α*-ASTREE model). The principal component analysis result of the e-tongue measurement showed that soy, coconut, and hazelnut PBMs had the most milk-like characteristics, with coconut being the most similar to whole milk and hazelnut to semi-skimmed milk.

On the other hand, Papp et al. [157] analyzed 111 samples of plant-based beverages (almond, cashew, oat, rice, coconut, soy, and spelt) by an e-nose (NeOse Pro model) and GC-MS to compare which methodology allowed the recognition of each raw material, as well as to see whether the brands could be distinguished between products of the same food matrix, based on the volatile compounds generated. Results showed that the e-nose, a novel methodology, allowed for more accurate differentiation between the main matrix, as well as between brands of the different matrix, in a cheaper way than the traditional methodology (GC-MS), indicating the potential of these new technologies to analyze food matrices accurately and at a lower cost.

Zhang et al. [158] also compared the e-nose with GC-MS, processing chickpea milk from the unroasted matrix. They subjected the samples to fermentation and compared the volatile profile by GC-MS and an e-nose (PEN3 model), mutually validating both methodologies. The results showed the ability of both methods to efficiently distinguish volatiles in all samples (both fermented and unfermented), although mutual validation of GC-MS with the e-nose showed that the e-nose was able to detect changes in the volatiles profile before and after the fermentation process. Continuing with vegetable probiotic beverages, Mu et al. [159] made a beverage fermented with walnut and purple rice, comparing it with milk fermented with the same strain (traditional yogurt). The fermented beverages were tested with an e-tongue (cTongue model), an e-nose (cNose model), as well as a panel of people with experience in sensory analysis. The performance of the e-tongue and e-nose was similar, based on the change in membrane potential produced by the substances to be analyzed, with an artificial sensory array. The analysis showed that the fermented yogurt possessed, in general, more sourness, while the fermented vegetable yogurt was sweeter. When all the results (e-tongue, e-nose, and expert panel) were compared together, it was observed that the taste score was better for the fermented vegetable drink than for the classic yogurt.

Finally, Pointke et al. [160] evaluated 15 marketed vegetable beverages (soy, oat, and almond) using an e-tongue (*α*-ASTREE model) and a panel of sensory analysis experts. The e-tongue analysis revealed few significant differences between products, making clear the importance of evaluating products according to the original matrix or raw material. Organic products were associated with negative attributes such as bitterness and astringency, especially in the case of soybeans. When comparing the e-tongue results with those obtained by the panel, the data showed a significant positive correlation for sweetness, sourness, and saltiness for all the beverages evaluated. Thus, an e-tongue could be used as a quick and easy tool for the sensory evaluation of basic flavors, in view of the correlation made with the expert panel. This would allow food manufacturers to develop products that match the sensory preferences of consumers of plant-derived analogues, reducing off-flavors. However, although these products may be attractive in terms of taste, they may not be recommended from a nutritional point of view due to their high content of sugars and additives because of the high scores obtained in bitterness and astringency parameters, and further research and development of analogues with a good sensory as well as nutritional profile should be pursued.

Altogether, current evidence demonstrates that plant-based dietary patterns offer environmental and nutritional benefits, despite sensory limitations remaining a significant challenge. MAs and plant-based beverages can facilitate the reduced consumption of conventional meat and animal-based drinks; however, research consistently identifies astringency and bitterness from plant proteins as key sensory barriers. Optimizing both protein type and concentration, as well as spice formulations, represents the primary approach to addressing these sensory deficits. In this sense, electronic sensory analysis has emerged as a critical evaluation tool with demonstrated reproducibility across multiple studies. The e-tongue methodology, employed in the majority of investigations, provides objective gustatory assessment with consistent results across the studies, as does the e-nose, which is as effective as traditional methodologies (GC-MS) in assessing the profile of volatile compounds related to flavors. These technologies offer rapid screening capabilities, enabling researchers to efficiently identify promising formulations before conducting resource-intensive consumer testing. Integrating electronic sensory technology as a complementary tool to traditional acceptance studies enables researchers to establish more efficient and evidence-based product development processes. This methodological approach may accelerate the formulation of sensorially acceptable plant-based alternatives, ultimately supporting broader adoption of sustainable food systems.

### 3.6. Strengths and Limitations

This study provides an integrative approach to multiple interconnected domains that are typically examined in isolation, offering valuable insights for future research in the field. However, this multidisciplinary scope, while representing a key strength, also presents certain limitations that must be acknowledged. This broad interdisciplinary coverage may limit the depth of analysis in individual areas compared to specialized reviews focusing on single domains. Although the authors have endeavored to synthesize the key findings and main conclusions with scientific rigor, more comprehensive analyses could be achieved through focused reviews of each field independently. Nevertheless, the primary value of this work lies precisely in its integrated examination of interconnected disciplines, enabling novel perspectives to guide future food science research with significant impact on market development, health impacts, consumer behavior, and environmental sustainability.

The temporal restriction to studies published between 2020 and 2025 aimed to focus on recent developments in this rapidly evolving field, as plant-based food technology and consumer acceptance patterns have undergone significant transformations in recent years. However, this restriction may have excluded some relevant studies from earlier periods. In terms of methodological evaluation, no systematic quality assessment tool was applied to evaluate the methodological rigor of the included studies, which represents a limitation in assessing the strength of evidence. However, the authors critically evaluated study quality based on established criteria within each respective field, enabling the synthesis of evidence from different disciplinary perspectives supporting the development of well-founded conclusions and evidence-based recommendations.

Additionally, the geographical distribution of the included studies shows potential bias, which is particularly relevant for sensory acceptance and consumer behavior findings, as food preferences, flavor profiles, and culinary traditions vary significantly across cultures. The sensory evaluation studies included were predominantly conducted in Western populations, whose taste preferences and food preparation methods may not be representative of global dietary patterns. Consequently, conclusions regarding consumer acceptance of plant-based alternatives may have limited generalizability to populations with different cultural food traditions and sensory expectations.

### 3.7. Critical Perspectives and Future Trends

Increasing the integration of plant-based and alternative proteins faces multifaceted barriers that extend beyond technological challenges alone. Production limitations remain substantial, particularly for novel protein sources where commercial-scale manufacturing presents significant hurdles. Economic constraints constitute another critical barrier, as evidenced by the premium pricing of many plant-based alternatives that limits accessibility across socioeconomic strata, while the substantial capital investments required for scaling novel food technologies create market entry barriers. Sensory acceptance limitations persist despite technological advances, with issues such as astringency, off-flavors, and textural deficiencies in plant proteins continuing to challenge consumer adoption, particularly among omnivorous populations. Most fundamentally, cultural limitations encompass deeply entrenched dietary traditions, food neophobia, and psychological barriers related to perceptions of naturalness and authenticity that transcend rational environmental or health considerations.

From a critical standpoint, cultural and psychological barriers likely represent the most intractable obstacles to widespread adoption, potentially outweighing technical or economic constraints in their resistance to change. The persistent gap between stated consumer intentions and actual purchasing behavior suggests that addressing sensory and cultural acceptance may be more pivotal than achieving perfect nutritional or environmental optimization. A particularly promising approach involves hybrid products that strategically combine animal and plant proteins, a strategy that may offer the most practical balance between sensory quality, consumer acceptance, and sustainability objectives. This approach abandons the pursuit of perfect meat replication while maintaining sensory familiarity and achieving meaningful environmental gains. Policy interventions emerge as essential catalysts, including targeted subsidies for plant protein research, taxation structures that internalize environmental costs of animal agriculture, and public procurement policies that normalize alternative proteins in institutional settings. Furthermore, although educational initiatives may help reposition these products, overcoming ingrained consumer perceptions of inferiority remains a persistent challenge, especially in cultures with strong meat traditions. The development of minimally processed, whole-food-based alternatives may prove more successful than highly engineered products, as they align better with consumer preferences for minimally processed foods while avoiding the concerns that increasingly influence food choices related to ultra-processed foods. A diet composed mainly of whole plant foods could, in turn, improve the environmental impact that food production has not only on the environment but also on human health. Again, it is important to emphasize the importance of enabling the acquisition of foods that are processed as little as possible by the overall population, because even if their origin is plant-based, the impact on health may be negative compared to that of whole plant foods.

Future trends in this field will likely be characterized by technological convergence and market segmentation strategies rather than universal solutions. Context-dependent approaches that account for regional agricultural practices, supply chain efficiency, and socio-cultural factors will become increasingly important, moving away from one-size-fits-all solutions toward more nuanced, localized strategies. Regulatory frameworks will mature to provide clearer pathways for novel food approval while establishing sustainability metrics that guide market development. Market evolution will likely favor product diversification over direct meat mimicry, with protein alternatives increasingly positioned as distinct food categories targeting specific use cases rather than universal meat replacements.

## 4. Conclusions

This review provided a panoramic and integrative analysis of plant-based foods and MSs, encompassing environmental sustainability, nutritional considerations, ingredient innovation, consumer acceptance, and analytical evaluation. By synthesizing interdisciplinary insights, this work addresses existing gaps in the literature, offering a holistic perspective on the current state and future potential of PBFs and MSs.

The findings underscore that PBFs and MSs present significant opportunities to mitigate the environmental impacts of conventional meat production, especially through reductions in GHG emissions, land use, and water consumption. Nutritionally, these alternatives provide some health advantages, such as lower saturated fat and no cholesterol, yet persistent issues with amino acid balance and micronutrient adequacy limit their capacity to fully replace meat nutritionally. Even so, the inclusion of these alternatives in dietary patterns could have a positive impact on health.

Innovations in ingredient sourcing, including the utilization of legumes, mycoproteins, and fermentation-derived components, have enhanced the sensory and nutritional profiles of PBFs and MSs. Nonetheless, consumer acceptance is influenced by several factors, such as taste, food neophobia, and price. Advanced analytical tools, such as electronic noses, electronic tongues, spectroscopy, and chemometric analyses, play a central role in product development and quality assurance, yet their integration into routine evaluation processes requires further advancement.

Despite the promising developments, limitations persist, including the need for long-term studies on health outcomes associated with PBF and MS consumption, and the need to address consumer skepticism regarding highly processed plant-based products. Future research should focus on optimizing the nutritional profiles of PBFs and MSs, enhancing sensory attributes to meet consumer expectations, and standardizing analytical methodologies for quality assessment. Interdisciplinary collaboration among food scientists, nutritionists, technologists, and behavioral scientists is essential to addressing these challenges and driving the evolution of plant-based MAs.

By providing a critical synthesis of current knowledge and identifying areas for improvement, this review aims to guide stakeholders, including researchers, industry professionals, and policymakers, in advancing the development and adoption of PBFs and MSs. Continued innovation and research are imperative to realizing the full benefits of these alternatives and to facilitating their integration into mainstream dietary practices, contributing to more sustainable and health-conscious food systems.

To conclude, while technological advances and analytical tools are steadily improving the sensory and nutritional quality of plant-based MSs, fully replicating the functional, emotional, and cultural roles of conventional meat remains an unresolved challenge. Bridging the gap between consumer ideals and actual purchasing behavior will require not only product optimization but also deeper insights into the social and psychological dimensions that shape food acceptance. A critical, interdisciplinary approach is therefore essential to guiding future innovation in this evolving field. However, a mixed approach, integrating plant-based and hybrid products, may currently offer the most practical balance between sensory quality, acceptance, and sustainability.

## Figures and Tables

**Table 1:** Main characteristics included studies on the health impact of plant-based diets.

Reference	*n*	Data Collection	Diet Classification	Follow-Up Years	Participant Age
Keaver et al. [50]	36,825	24 h dietary recall	Comprehensive diet quality index:	8.3	47.1 ± 0.2 (average)
			Overall (cDQI)		
			Animal-based (aDQI)		
			Plant-based (pDQI)		
Chen et al. [51]	13,154	Food frequency questionnaire	Plant-based index:	5.7	86.9 ± 11.4 (average)
			Overall (oPDI)		
			Healthy (hPDI)		
			Unhealthy (uPDI)		
Li et al. [52]	40,074	24 h dietary recall	Plant-based index:	7.8	47.3 ± 19.4 (average)
			oPDI		
			hPDI		
			uPDI		
Weston et al. [53]	3635	Food frequency questionnaire	Plant-based index:	15	21–95
			oPDI		
			hPDI		
			uPDI		
Wang et al. [54]	11,939	24 h dietary recall	Plant-based index:	11.2	49 (average)
			oPDI		
			hPDI		
			uPDI		
			Healthy eating index (HEI-2015)		
			Pro-vegetarian diet index (PVD)		
Zhou et al. [55]	189,003	24 h dietary recall	Plant-based index:	9.6	55.99 ± 7.95 (average)
			oPDI		
			hPDI		
			uPDI		
Yuan et al. [56]	2675	Food frequency questionnaire	Plant-based index:	10	102.33 (average)
			oPDI		
			hPDI		
			uPDI		
			Healthy plant-based foods index (HPF)		
			Unhealthy plant-based foods index (uHPF)		
			Animal-based foods index (AF)		
Kim et al. [57]	144,729	Food frequency questionnaire	Plant-based index:	21	45–75
			oPDI		
			hPDI		
			uPDI		
Abris et al. [58]	88,400	Food frequency questionnaire	Vegetarians:	11.07	≥30
			Vegans		
			Pescovegetarians		
			Lacto-ovovegetarians		
			Non-vegetarians		
Oncina-Cánovas et al. [59]	597	Food frequency questionnaire	Pro-vegetarian dietary pattern:	12	≥65
			Overall (oPVG)		
			Healthy (hPVG)		
			Unhealthy (uPVG)		
Huang et al. [60]	7843	Food frequency questionnaire	Plant-based index:	11	82.2 ± 10 (average)
			oPDI		
			hPDI		
			uPDI		

*n*: Total number of subjects included in each study.

**Table 2:** Risk (Hazard Ratio or Odds Ratio) of all-cause mortality by type of dietary pattern classification observed in each study selected.

Reference	Dietary Pattern Classification															
	oPDI	hPDI	uPDI	oPVG	hPVG	uPVG	AF	uHPF	HPF	Veg.	Non-Veg.	cDQI	aDQI	pDQI	PVD	HEI-2015
Keaver et al. [50]	-	-	-	-	-	-	-	-	-	-	-	0.75	n.a.	0.66	-	-
Chen et al. [51]	0.92	0.81	1.17	-	-	-	-	-	-	-	-	-	-	-	-	-
Li et al. [52]	0.80	0.86	1.33	-	-	-	-	-	-	-	-	-	-	-	-	-
Weston et al. [53]	1.07	0.94	1.15	-	-	-	-	-	-	-	-	-	-	-	-	-
Wang et al. [54]	n.a.	n.a.	n.a.	-	-	-	-	-	-	-	-	-	-	-	n.a.	0.87
Zhou et al. [55]	0.87	0.92	1.29	-	-	-	-	-	-	-	-	-	-	-	-	-
Yuan et al. [56]	0.81	0.79	1.10	-	-	-	1.17	0.95	0.81	-	-	-	-	-	-	-
Kim et al. [57]	0.85 (male) 0.89 (female)	0.88 (male) 0.86 (female)	1.03 (male) 1.11 (female)	-	-	-	-	-	-	-	-	-	-	-	-	-
Abris et al. [58]	-	-	-	-	-	-	-	-	-	0.89	1 (reference)	-	-	-	-	-
Oncina-Cánovas et al. [59]	-	-	-	0.85	0.90	1.53	-	-	-	-	-	-	-	-	-	-
Huang et al. [60]	1.10 (highest increase) 1.32 (highest decrease)	0.96 (highest increase)	1.13 (highest increase)	-	-	-	-	-	-	-	-	-	-	-	-	-
		1.21 (highest decrease)	0.90 (highest decrease)													

Gray (-): Not evaluated; yellow (n.a.): no association observed; red (HR/OR): higher risk of mortality; green (HR/OR): lower risk of mortality; white (HR/OR): control or reference. oPDI: overall plant dietary index; hPDI: healthy plant dietary index; uPDI: unhealthy plant dietary index; oPVG: overall pro-vegetarian dietary pattern; hPVG: healthy pro-vegetarian dietary pattern; hPVG: unhealthy pro-vegetarian dietary pattern; AF: animal-based foods index; uHPF: unhealthy plant-based foods index; HPF: healthy plant-based foods index; Veg.: vegetarians; Non-Veg.: non-vegetarians; cDQI: overall comprehensive diet quality Index; aDQI: animal comprehensive diet quality index; pDQI: plant-based comprehensive diet quality Index; PVD: pro-vegetarian diet index; HEI-2015: healthy eating index.

**Table 3:** Environmental impacts of foods.

Food Type	GHG Emissions (kg CO_2_ eq/kg)	Land Use (m^2^/kg)	Water Use (L/kg)
Beef	27.0	326	15,415
Pork	12.0	70	5988
Chicken	6.9	12	4325
Soy	2.0	4	2145
Lentils	0.9	2.5	1250
Vegetables	0.6	1.5	500
Insects	5.7	1.9	247
Mycoprotein	5.3	1.5	4996
Cultured meat	5.7	1.9	414
Microalgae	81.6	3.1	53,944

Adapted from Aimutis & Shirwaiker [37], Prescott et al. [47], Harwatt et al. [18], and Mazac et al. [25].

## Data Availability

No new data were created or analyzed in this study. Data sharing is not applicable to this article.

## Abbreviations

PBF	Plant-Based Food
MS	Meat Substitute
MA	Meat Analogue
GHG	Greenhouse Gas
LCA	Life Cycle Assessment
PRISMA	Preferred Reporting Items for Systematic Reviews and Meta-Analyses
SSPs	Shared Socioeconomic Pathways
FFQ	Food Frequency Questionnaire
HR	Hazard Ratio
OR	Odds Ratio
CRP	C-Reactive Protein
PDI	Plant-Based Diet Index
oPDI	Overall Plant-Based Diet Index
hPDI	Healthy Plant-Based Diet Index
uPDI	Unhealthy Plant-Based Diet Index
HPF	Healthy Plant-Based Foods Index
uHPF	Unhealthy Plant-Based Foods Index
AF	Animal-Based Foods Index
oPVG	Overall Pro-Vegetarian Dietary Pattern
hPVG	Healthy Pro-Vegetarian Dietary Pattern
uPVG	Unhealthy Pro-Vegetarian Dietary Pattern
HEI-2015	Healthy Eating Index–2015
PVD	Pro-Vegetarian Diet Index
DQI	Diet Quality Index
aDQI	Animal-Based Diet Quality Index
cDQI	Comprehensive Diet Quality Index
pDQI	Plant-Based Diet Quality Index
EPA	Eicosapentaenoic Acid
DHA	Docosahexaenoic Acid
SFA	Saturated Fatty Acid
MUFA	Monounsaturated Fatty Acid
PUFA	Polyunsaturated Fatty Acid
e-nose	Electronic Nose
e-tongue	Electronic Tongue
e-eye	Electronic Eye
GC-MS	Gas Chromatography–Mass Spectrometry
SPC	Sustainable Production and Consumption (journal context)
AFW	Agri-Food Waste
